# The transcriptional programme controlled by Runx1 during early embryonic blood development

**DOI:** 10.1016/j.ydbio.2012.03.024

**Published:** 2012-06-15

**Authors:** Yosuke Tanaka, Anagha Joshi, Nicola K. Wilson, Sarah Kinston, Shinichi Nishikawa, Berthold Göttgens

**Affiliations:** a Laboratory for Stem Cell Biology, RIKEN Center for Developmental Biology, Kobe, Japan; b Department of Haematology, University of Cambridge, Cambridge Institute for Medical Research, Cambridge, UK

**Keywords:** Haematopoiesis, Transcriptional control, Early blood development, Runx1, ChIP-Seq, Gene expresssion

## Abstract

Transcription factors have long been recognised as powerful regulators of mammalian development yet it is largely unknown how individual key regulators operate within wider regulatory networks. Here we have used a combination of global gene expression and chromatin-immunoprecipitation approaches during the early stages of haematopoietic development to define the transcriptional programme controlled by Runx1, an essential regulator of blood cell specification. Integrated analysis of these complementary genome-wide datasets allowed us to construct a global regulatory network model, which suggested that key regulators are activated sequentially during blood specification, but will ultimately collaborate to control many haematopoietically expressed genes. Using the CD41/integrin alpha 2b gene as a model, cellular and *in vivo* studies showed that CD41 is controlled by both Scl/Tal1 and Runx1 in fully specified blood cells, and initiation of CD41 expression in E7.5 embryos is severely compromised in the absence of Runx1. Taken together, this study represents the first global analysis of the transcriptional programme controlled by any key haematopoietic regulator during the process of early blood cell specification. Moreover, the concept of interplay between sequentially deployed core regulators is likely to represent a design principle widely applicable to the transcriptional control of mammalian development.

## Highlights

► Genome-scale analysis of Runx1 targets during early blood development. ► Integrated analysis of ES-cell and early mouse embryo expression profiles. ► Runx1 is a critical regulator of CD41 expression in early embryos. ► Connecting sequentially-activated regulators stabilises emerging networks.

## Introduction

To enhance our understanding of metazoan development, it will be critical to both identify cellular intermediates en route from the pluripotent stem cell to fully differentiated mature lineages, as well as to discover the molecular mechanisms which control individual state transitions as cells journey down a given differentiation trajectory. The development of blood cells from pluripotent embryonic stem cells has served as an important model system (Keller et al., 2002) with several key intermediate cellular stages and major regulatory genes now known (Ottersbach et al., 2010). The Ets family transcription factor Etv2 for example is required for the differentiation of early mesodermal cells into haemangioblasts (Sumanas and Lin, 2006; Lee et al., 2008), an intermediate cell population with both haematopoietic and endothelial potential (Choi et al., 1998). The bHLH transcription factor Scl (also known as Tal1) is subsequently required for the maturation of haemangioblasts into haemogenic endothelial cells (Lancrin et al., 2009), an intermediate cell population with endothelial morphology yet a strong propensity for differentiation into haematopoietic cells. This latter maturation step is controlled by the Runt domain transcription factor Runx1, and importantly this endothelial-to-haematopoietic transition has been shown to require Runx1 activity both in ES cell differentiation as well as mouse embryo *in vivo* studies (Chen et al., 2009; Lancrin et al., 2009).

Despite the accumulating knowledge on key intermediate cellular stages and major regulators, our understanding of the molecular mechanisms that control the important differentiation state transitions during haematopoietic specification remains rudimentary. Since many of the early “decision makers” are transcription factors, identification of their target genes represents a potentially very direct route into deciphering the molecular and cell biological processes underlying haematopoietic cell fate specification. Target gene identification has traditionally been approached by performing gene expression profiling experiments comparing wild type with mutant cells. This approach however does not readily distinguish direct from indirect targets. Chromatin immunoprecipitation (ChIP) experiments identify regions in the genome bound by a given transcription factor, and therefore have the capacity to establish direct regulatory relationships between an upstream transcription factor and its target gene. The recent coupling of ChIP experiments with high-throughput sequencing (ChIP-Seq) has made comprehensive genome-scale identification of transcription factor target genes a realistic undertaking (Barski et al., 2007; Johnson et al., 2007; Robertson et al., 2007). Indeed, ChIP-Seq analysis has now been performed for several key haematopoietic regulators (Cheng et al., 2009; Fujiwara et al., 2009; Wilson et al., 2009, 2010a; Yu et al., 2009; Heinz et al., 2010; Kassouf et al., 2010; Lin et al., 2010; Soler et al., 2010; Li et al., 2011). Importantly however, no reports so far have analysed genome-wide binding patterns in early developmental cell populations. Since ChIP-Seq studies of the same factor in different cell types have shown that binding patterns are largely cell-type specific (Hannah et al., 2011; Palii et al., 2011), it is imperative that such analysis will be performed in early developmental cell populations in order to enhance our understanding of the molecular control of early cell fate specification.

Here we have defined the transcriptional programme controlled by Runx1 during early stages of blood development, using embryonic stem (ES) cell differentiation as a model system. Integrated analysis of expression and ChIP-Seq profiling datasets identified candidate direct Runx1 target genes, where those targets that correlated with Runx1 expression were enriched for biological functions related to haematopoietic development. By contrast, those candidate target genes that were negatively correlated with Runx1 expression were enriched for genes affiliated with non-haematopoietic tissue development. Moreover, integrated analysis of our timecourse data with a newly generated expression dataset for E7.5 Runx1^−/−^ embryos suggested a model whereby core regulatory circuits are activated sequentially during early development, but then function collectively within wider regulatory networks in fully specified blood cells.

## Material and methods

### Cell culture and *in vitro* differentiation of ES cells

Runx1^Venus/+^ ES cells were cultured and differentiated on OP9 cells as described (Hirai et al., 2005). The following mAbs were used for cell sorting: APC–anti-Flk1 (AVAS12), biotin–anti-VE-cadherin (VECD1; followed by streptavidin–PE–Cy7), and PE–anti–CD41 (MWreg30; eBioscience). AVAS12 and VECD1 were prepared and labelled in our laboratory. Cell staining was performed as previously described (Hirai et al., 2005) and dead cells excluded by propidium iodide staining. Runx1^SACRE/LacZ^ ES cells in which a Runx1-LacZ allele (Tanaka et al., 2012) can be reactivated by 4-hydroxy-tamoxifen (4OHT) were generated from E2.5 morulas as described (Bryja et al., 2006; Samokhvalov et al., 2006). Maintenance and differentiation of Runx1^SACRE/LacZ^ ES cells was performed in the same manner as for the Runx1^Venus/+^ ES cells. Following differentiation on OP9 feeder cells, Flk1^+^ cells were collected on day 4 by cell sorting, and then recultured on Collagen type IV dishes with or without 1 μM 4OHT. After 24 h- and 48 h-culture, all cells were harvested and collected for RNA isolation.

### Haematopoietic and endothelial differentiation potential assay

For haematopoietic progenitor (HPC) colony-forming assays, 1000 cells were recultured in methylcellulose media MethoCult GF M3434 (StemCell Technologies). For endothelial differentiation, 1000 cells were recultured on OP9 in the presence of 25 ng/ml recombinant human vascular endothelial growth factor (rhVEGF). Endothelial colonies were determined by immunostaining using anti-CD31 antibody (390) (eBioscience). To monitor B-cell and myeloid differentiation, 500 cells per well were seeded on OP9 monolayer and cultured in α-MEM supplemented with 10% FCS, 50 μM 2-ME, 10 ng/ml IL-7 and 10 ng/ml Flt3L in 24 well dishes. The supernatant was collected and analysed by FACS Aria after 14 days. CD19 and Mac1 were used to detect B cells and myeloid cells, respectively.

### Embryo experiments

E7.5 and E8.5 embryos were dissociated using 0.25% trypsin/EDTA, and single cell suspensions were stained by the indicated antibodies followed by FACS analysis using antibodies against cell surface markers as well as FDG-staining for β-GAL activity. Data were analysed using FACSDiva (BD Bioscience) and FlowJo (Tree Star, Ashland, OR, USA). For microarray analysis FDG/VE-cadherin double positive cells were sorted from E7.5 Runx1^LacZ/+^ and E7.5 Runx1^LacZ/LacZ^ embryos, respectively. Cells were lysed in RLT buffer (Qiagen). RNA was purified using RNeasy micro elute column (Qiagen). Microarray analysis was performed using the SurePrint G3 Mouse GE platform (Agilent Technologies). For whole mount immunostaining, E7.5 Runx1^LacZ/+^ and E7.5 Runx1^LacZ/LacZ^ embryos were fixed by 2% PFA/PBS on ice for 20 min. After washing with PBS, samples were blocked with PBST/2% skimmed milk/1% normal donkey serum overnight. Primary antibody used was anti-CD41 (MWReg30) and secondary antibody used was donkey anti-rat IgG Alexa647 (Invitrogen).

### Quantitative RT-PCR analysis

Cells were lysed in RLT buffer (Qiagen). RNA was purified using RNeasy micro elute column (Qiagen) and used for reverse transcription by SuperScriptIII (Invitrogen). Real time- PCR was performed by PowerSyber Green kit (ABI) using ABI 9700. The following oligonucleotide primers were used for q-PCR: Gfi1b, FW CAGGATGGGGAATCACCACTC, REV GGGGTCTGTGTGTAGCTGT; Cldn5, FW GCAAGGTGTATGAATCTGTGCT, REV GTCAAGGTAACAAAGAGTGCCA; Gata1, FW CAGAACCGGCCTCTCATCC, REV TAGTGCATTGGGTGCCTGC; bH1, FW GAAACCCCCGGATTAGAGCC, REV GAGCAAAGGTCTCCTTGAGGT; bMajor, FW GACCCAGCGGTACTTTGATAGC, REV TGAGGCTGTCCAAGTGATTCA; Runx1, FW GCAGAACTGAGAAATGCTACCG, REV GCAACTTGTGGCGGATTTGTA.

### Gene expression profiling and data processing

Gene-expression profiling of VE-cadherin^+^/LacZ^+^ cells from E7.5 Runx1^WT/LacZ^ or Runx1^LacZ/LacZ^ embryos was performed using Agilent SurePrint G3 Mouse GE microarrays. For each sample 2 biological and 2 technical replicates were generated making 8 samples in total for analysis. Details regarding sample preparation, hybridisation, and image acquisition are as follows: Briefly, VE-cadherin^+^/LacZ^+^ cells from E7.5 Runx1^WT/LacZ^ or Runx1^LacZ/LacZ^ embryos were enriched/sorted by FACSAria. Total RNA was isolated using Qiagen RNeasy Micro Kit. cDNA preparation, *in vitro* transcription, labelling, hybridisation, washing, and staining steps were performed according to standard Agilent protocols. Probes showing 1.5 fold change with *P*-value<0.05 were considered differentially expressed. Genome-wide expression profiles of 4 samples (Runx1^−^VE^+^, Runx1^+^VE^+^CD41^−^, Runx1^+^VE^+^CD41^+^ and Runx1^+^VE^−^CD41^+^) were collected using Affymetrix Mouse Genome 430 2.0 microarrays, and a second replicate sample set was analysed using Agilent SurePrint G3 Mouse GE microarrays.

**Fig. 1:**
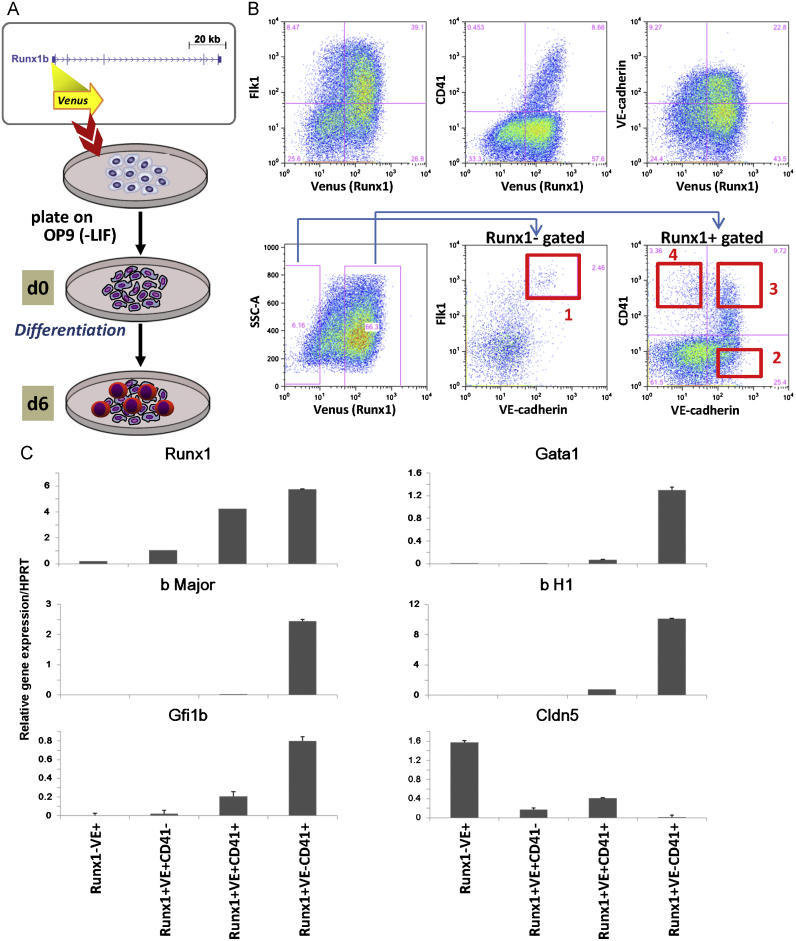
Isolation of cell populations and validation by gene expression analysis. (A) ES cell differentiation using an ES cell line model containing a Venus reporter gene knocked into one of the endogenous Runx1 alleles. The ES cells were plated onto OP9 cells without LIF and differentiated for 6 day. (B) FACS plots from day 6 Runx1^venus/+^ ES cells differentiated on OP9. Purification of four cell populations of increasing haematopoietic potential using flow cytometry (1) Runx1^−^VE^+^Flk1^+^, (2) Runx1^+^VE^+^CD41^−^, (3) Runx1^+^VE^+^CD41^+^ and (4) Runx1^+^VE^−^CD41^+^. (C) Populations 1–4 were validated using quantitative RT-PCR analysing the expression of Runx1, Gata1, bMajor, bH1, Gfi1b and Cldn5.

Details regarding sample preparation, hybridisation, and image acquisition are as follows: Briefly, 1×10^5^ undifferentiated ES cells were cultured on confluent OP9 cell layers in 10 cm dishes to induce differentiation. After 6 day of ES cell differentiation, cultured cells were harvested for FACS analysis and sorting. cDNA preparation, *in vitro* transcription, labeling, hybridisation, washing, staining and image processing steps were performed according to standard Affymetrix and Agilent protocols. Quality control consisted of visual inspection of the array image for artifacts, assessment of RNA degradation plots, and inspection of rank versus residual plots after normalisation and probe set summarisation. A total of 7753 genes showed dynamic expression patterns (standard deviation across 4 conditions>1.0 and expression level >100 in at least one sample). Expression dynamics were visualised using GEDI plots (Eichler et al., 2003). Correlation with Runx1 expression was determined using Pearson correlation. We analysed the datasets generated by 2 platforms (Affymetrix and Agilent) individually, and only considered the genes correlated or negatively correlated with Runx1 in both datasets. Expression levels of 6 genes were validated by Q-RT-PCR (Fig. 1C).

### ChIP-Seq analysis

Three million cells were purified by cell sorting for each population. ChIP assays were performed as described (Wilson et al., 2010b) using Runx1 antibody ab23980-100 from Abcam. Reads were mapped to the mouse genome build mm9 using bowtie (Langmead et al., 2009). Custom tracks, peak positions and candidate target gene lists are available from our website (http://hscl.cimr.cam.ac.uk/Runx1-ChIP2011/Runx1-ChIP2011.html). Peaks were called using MACS (Zhang et al., 2008) (paramenters:—mfold=16, —tsize=35, —bw=100, —*P*-value=1e^−9^, —gsize=2200000000) using the IgG sample as control, which identified 227, 195 and 1143 peaks. We further selected high confidence peaks by excluding peaks in repeat regions resulting into 67, 51 and 747 peaks in the 3 populations (Runx1^+^VE^+^CD41^−^, Runx1^+^VE^+^CD41^+^, Runx1^+^VE^−^CD41^+^). Saturation curves were generated to assure that the sequencing depth was sufficient (Supplementary Fig. 1). The correlation coefficients of peak heights between the three populations illustrate that populations 2 and 3 have very similar binding patterns (Pearson’s correlation coefficient>0.80) providing a rationale to split the peaks into 2 partitions (2,3,4 and 4 only respectively; Supplementary Fig. 2). For motif analysis, the middle 200 bp was taken and peaks with more than 60% repetitive sequence excluded. MEME (Bailey and Elkan, 1994) was used for *de novo* motif discovery with standard settings on the combined peaks from all three populations as well as the individual populations. Matches to consensus sequences were determined as described using TFBSSearch (Chapman et al., 2004). Fig. 4 tabulates all overrepresented sequence motifs on Runx1 peaks from all 3 populations. Intragenic and promoter (within 500 bp from TSS) binding events were associated to the corresponding gene while intergenic binding events were mapped to the nearest 5′ and 3′ genes within 50 kb. Supplementary Fig. 3 contains a detailed analysis of the three individual groups of peaks with respect to motif search and GO functional enrichment analysis. As populations 2 and 3 both showed relatively small numbers of binding events with a very high overlap between them as well as with population 4, population 2 and 3 peaks were merged and referred to as ‘2,3,4’ and peaks unique to population 4 referred as ‘4 only’. Genes up- or down-regulated by Runx1 overexpression in hemangioblasts were obtained from (Sakai et al., 2009). Supplementary Table 1 provides all 787 peaks with peak heights in 3 samples as well Tal1, Gata2 and Fli1 binding in HPC cells.

### Validation of CD41 as a Runx1 target

The CD41 promoter (863 bp) was amplified from mouse DNA (primers AGTGACTCCGTCCACAAACA and TAGGACGTTTGGGAAGAAGG), inserted upstream of luciferase in pGL2 and assayed as described (Landry et al., 2008). E7.5 embryos were fixed for 5 min in 2% PFA on ice, and dehydrated in increasing concentrations of methanol/PBS. Endogenous peroxidase activity was blocked by H_2_O_2_ treatment. After rehydration, embryos were stained with anti-CD41, followed by goat-anti-rat Ig-HRP (Jackson ImmnoResearch). Signals were detected using the Peroxidase Substrate Kit DAB (Vector Laboratories).

### Accession numbers

Expression array and ChIP-Seq data have been submitted to the NCBI Gene Expression Omnibus (accession number GSE29112).

## Results

### Many genes dynamically expressed during early blood specification correlate with Runx1 expression

To gain new insights into the process of early haematopoietic development from multipotential mesodermal precursor cells, it will be important to gain access to purified cell populations with increasing levels of haematopoietic commitment. To this end, we decided to take advantage of ES cell differentiation systems based on an ES cell line model that contains a Venus fluorescent reporter gene knocked into one of the endogenous Runx1 alleles (see Fig. 1A). Of note, even though the timing of haematopoietic stem cell (HSC) development is slightly altered in Runx1^+/−^ embryos (Cai et al., 2000), the stem and progenitor cells generated are functional. Like other transcription factor knock-ins with phenotypes in heterozygous mice such as Scl and Brachyury (Stott et al., 1993; Curtis et al., 2004; Lacombe et al., 2010), the Runx1^Venus/+^ ES cells therefore represent an effective means to purify cell populations of interest, which are otherwise not accessible. Following *in vitro* differentiation, cells can be purified by flow cytometry based on their levels of Venus fluorescence as a surrogate marker for Runx1 expression, and through the use of antibodies against cell surface markers can be further fractionated into cell populations with defined biological properties.

As shown in Fig. 1(B), Runx1^Venus/+^ ES cells expressed haematopoietic (CD41) and endothelial (VE-cadherin) lineage markers following 6 days of OP9 co-culture. Thus we decided to use this approach to purify four cell populations: Population 1 contained Runx1-Venus negative/VE-cadherin positive cells, a population recognised as being committed to the endothelial lineage. Population 2 contained Runx1-Venus positive/VE-cadherin positive/CD41 negative cells, corresponding to lateral mesodermal progenitors with both endothelial and haematopoietic differentiation potential. Population 3 contains Runx1-Venus positive/VE-cadherin positive/CD41 positive cells, and thus represents a population more committed to the haematopoietic lineage, but still retaining some endothelial characteristics. Population 4 contains Runx1-Venus positive/VE-cadherin negative/ CD41 positive cells which represent haematopoietic progenitor cells. To further corroborate these phenotypic characteristics, cells from each population were isolated by flow cytometry and analysed by performing colony forming assays and studying the expression of marker genes. Comprehensive Q-RT-PCR analysis demonstrated specific expression of erythroid genes only in population 4, and downregulation of the endothelial marker claudin 5 in populations 2–4 (Fig. 1C).

**Fig. 2:**
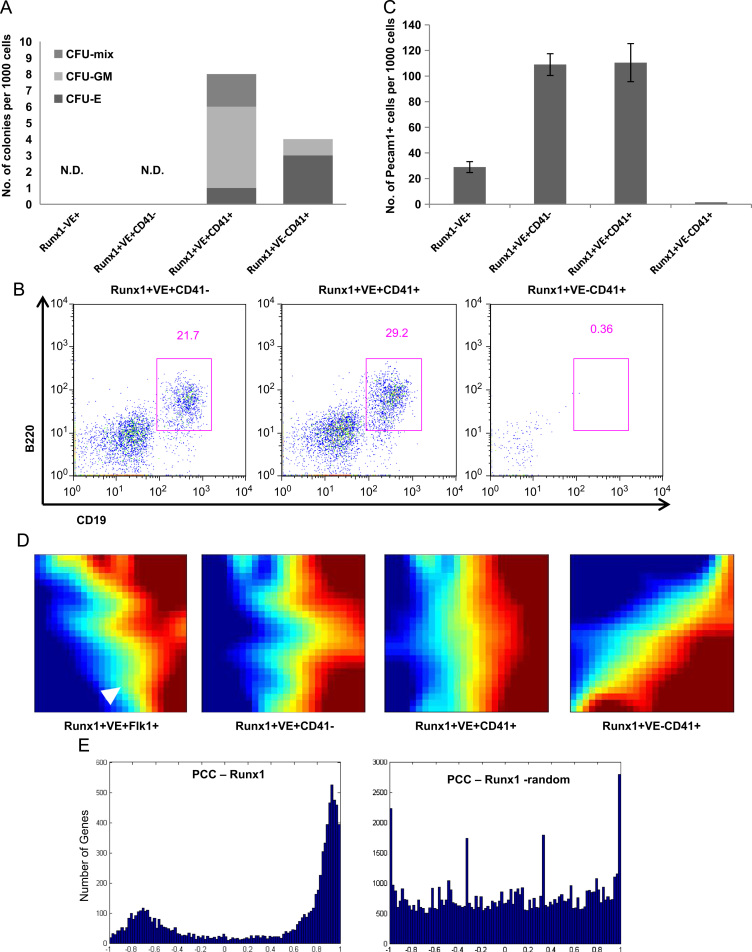
Endothelial and haematopoietic potentials of the day 6 differentiated Runx1^Venus/+^ ES cell populations and gene expression dynamics during early blood specification. (A) The 4 sorted populations were cultured in methylcellulose. At day 10 of culture, haematopoietic colonies were counted. (B) Lymphoid potential of the 3 Runx1 expressing populations. The 3 Runx1 expressing populations were sorted and cultured on OP9 in the presence of 10 ng/ml IL-7 and Flt3L for 14 day. Non-adherent cells were collected from the culture and analysed by flow cytometry for the expression of B220 and CD19. (C) Endothelial cell colony forming potential of the 4 populations. The 4 populations were sorted and cultured on OP9. Endothelial colonies judged by immunostaining against Pecam1 were counted after 4 day. (D) Global analysis of changes in gene expression profiles of the four sorted samples represented using gene expression dynamics investigator (GEDI (Eichler et al., 2003)) plots with a colour gradient (blue representing lowly expressed genes to red representing highly expressed genes). The white arrowhead indicates the position of Runx1, the expression of which increases across the four samples as expected. (E) Correlation analysis of dynamically expressed genes in relation to Runx1 expression; (i) Pearson’s correlation coefficients (PCC) for all dynamically expressed genes with respect to Runx1 showing that most of these genes correlate positively or negatively with Runx1 expression. (ii) Control analysis with randomised samples confirming that the coherent dynamic transcriptome with respect to Runx1 is valid.

To further investigate the haematopoietic and endothelial potentials of these 4 populations, sorted cells were cultured in methylcellulose medium to investigate haematopoietic progenitor cell (HPC) colony-forming potential. As shown in Fig. 2(A), populations 1 and 2 (Runx1^−^/VE-cadherin^+^ and Runx1^+^VE-cadherin^+^CD41^−^) had no haematopoietic potential using this assay. By contrast, both populations 3 and 4 (Runx1^+^/VE-cadherin^+^/CD41^+^ and Runx1^+^/VE-cadherin^−^/CD41^+^) had colony-forming potential, but CFU-mix colonies were detected only in population 3 (Runx1^+^VE-cadherin^+^CD41^+^). Next we examined lymphoid differentiation potential of the three 3 Runx1^+^ populations by reculturing cells on an OP9 stromal layer in the presence of IL-7 and Flt3L for 14 days. CD19^+^B220^+^ cells were detected in the two Runx1^+^VE-cadherin^+^ populations (populations 2 and 3) (Fig. 2B), suggesting that only those two populations have definitive haematopoietic potential. Population 2 had no haematopoietic potential in methylcellulose medium, but could generate B lymphoid cells in OP9 culture, thus suggesting that these cells require OP9-support to differentiate into the haematopoietic lineage, a notion consistent with previous observations (Hashimoto et al., 2007). We also examined the endothelial potential of all 4 populations using reculture of sorted cells on OP9 in the presence of VEGF. After 4 days of culture, immunostaining using anti-Pecam antibody revealed Pecam^+^ colonies only using cells from the three VE-cadherin^+^ populations (populations 1– 3; see Fig. 2(C)). Taken together, these data support a model whereby population 1 (Runx1^−^/VE-cadherin^+^) is likely restricted to endothelial potential, populations 2 and 3 have both definitive haematopoietic (lymphoid) and endothelial potential and population 4 appears restricted to primitive haematopoietic (erythroid/myeloid) potential.

To obtain insights into the global transcriptional programmes characteristic for these four early developmental cell populations, we next performed gene expression profiling on all four populations. When interrogated across the four different populations, over 6000 genes displayed dynamic expression patterns. Analysis using the Gene Expression Dynamics Inspector (GEDI) tool (Eichler et al., 2003) demonstrated that dynamically expressed genes largely fall into two categories, those that are progressively downregulated and those that are upregulated when the four populations are arranged from population 1 to 4 (see Fig. 2D). As expected, Runx1 itself was amongst the upregulated genes (see white arrowhead in left-hand panel of Fig. 2D) thus providing internal consistency between endogenous Runx1 expression and the cell sorting strategy based on the knock-in transgene.

To define all genes whose dynamic expression profile across the four populations might be under the control of Runx1, we next calculated Pearson correlation coefficients for all dynamically expressed genes in relation to Runx1. As shown in Fig. 2(E), the majority of these genes showed either positive correlation or negative correlation with Runx1 expression, and was thus consistent with Runx1-mediated activation and repression, respectively. However, inference based on correlated gene expression alone is unable to distinguish direct regulation from indirect regulation or indeed simple correlation without any regulatory linkage. It is unlikely that Runx1 on its own is responsible for virtually all of the gene expression dynamics seen across the four populations analysed here, thus suggesting that additional approaches would be required to identify the subset of genes directly controlled by Runx1.

### Genome-wide analysis demonstrates dynamic recruitment of Runx1 protein to its target sites during haematopoietic specification

Computational analysis had suggested that transcriptional datasets alone would not be sufficient to identify the transcriptional programme downstream of Runx1 during early haematopoietic specification. The direct demonstration of Runx1 protein binding to regulatory regions within candidate target gene loci would provide highly complementary information, which can be obtained at genome-scale by ChIP-Seq. We therefore purified 3 million cells from each of the Runx1 expressing populations 2–4 (Fig. 1D), prepared chromatin and performed ChIP experiments using an antibody against endogenous Runx1 protein. A parallel ChIP assay was also performed on 10 million unsorted cells using a non-specific IgG antibody to serve as a negative control for subsequent analysis of Runx1 binding.

**Fig. 3:**
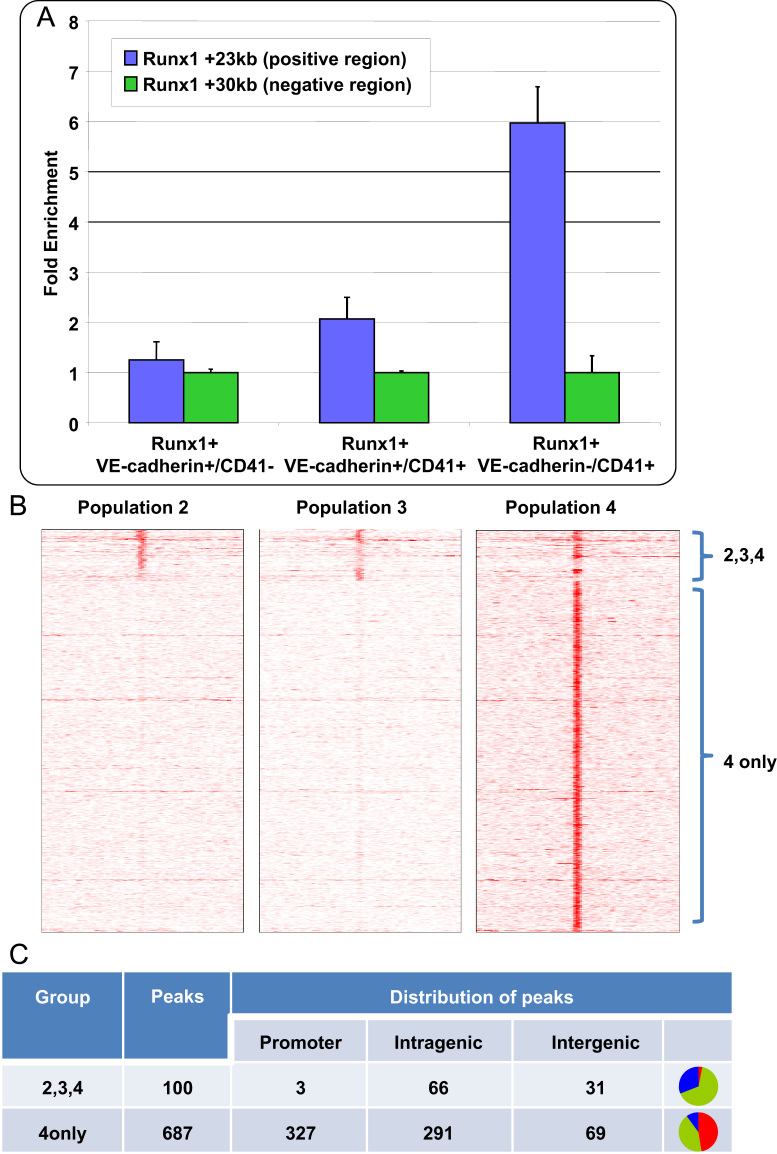
ChIP-Seq analysis of populations 2–4 permits genome-wide analysis of Runx1 recruitment to its target sites during early haematopoietic development. (A) Validation of Runx1 ChIP assays. Runx1 ChIP assays in populations 2–4 were validated using quantitative PCR with primer pairs for a region known to be bound by Runx1 (+23 kb, blue bars) and a negative control region (+30 kb, green bars) (Wilson et al., 2009). (B) Heatmaps displaying density profiles of Runx1 binding in populations 2–4 to all 787 peak regions, with the peak summit at the center shown with 5 kb flanking sequence either side. Few peaks occur in populations 2 and 3 with most peaks showing strong binding in population 4. (C) Analysis of peak locations with respect to genes. Peaks shared by all populations are rarely found in promoters whereas peaks found in population 4 are equally distributed between promoters and intragenic regions. The pie charts on the right show proportion of peaks in promoters (red), intragenic (green) and intergenic (blue).

Analysis of ChIP material by quantitative real-time PCR demonstrated significant binding of Runx1 protein to the Runx1 +23 enhancer (Nottingham et al., 2007) in cell population 4, with less binding in population 3 (See Fig. 3A). We therefore proceeded to analyse all three Runx1 ChIPs as well as the IgG negative control sample by high-throughput sequencing using the Illumina Genome Analyzer II platform. DNA sequencing of ChIP material provided 13.4 million, 9.8 million, 16.6 million and 9.3 million uniquely mappable reads for populations 2, 3, 4 and the IgG control sample, respectively, thus demonstrating that sufficient sequencing depth had been achieved to analyse genome-wide binding patterns of Runx1.

To permit genome-wide analysis of regions bound by Runx1 protein, binding peaks were identified using software tools designed to take advantage of the negative control sample for elimination of false positive peaks of enrichment (see Material and methods). A total of 787 regions were identified to be bound by Runx1 in at least one of the three samples, with most peaks predicted to only be present in population 4 (see Supplementary Table 1). To further explore these results, Runx1 binding across all 787 peak regions was visualised using heat-maps displaying the strength of binding across 10 kb segments centred on each peak region (see Fig. 3B). Heat map analysis indeed supported a model whereby Runx1 binding in the earlier populations is absent or highly transient except for a small subset of peaks, with the large majority of regions only showing robust binding in population 4.

In order to further validate the 787 Runx1-bound regions defined above, we compared them to Runx1 binding events previously mapped in the haematopoietic progenitor cell line HPC7 (Wilson et al., 2010a). 37% of the new peaks coincided with regions also bound by Runx1 in the HPC7 cell line (Supplementary Fig. 4), thus providing additional support for the validity of the new data. Moreover, of 839 Runx1 candidate target genes identified by Hollenhorst et al. (2009) in the human leukaemia cell line Jurkat , 503 overlapped with the candidate Runx1 target genes defined here, thus demonstrating a very significant overlap (*P*-value −1.2e^−10^) (Supplementary Fig. 6). In order to compare Runx1 levels in the ES-cell derived populations 1–4 with the HPC7 cell line, we performed a ChIP assay on the HPC7 cells using the same conditions as used for the ES-derived populations, and then performed quantitative comparisons by Q-PCR. Even the ES-cell derived population with the highest Runx1 binding (e.g., population 4) consistently displayed only about half as much Runx1 binding as the HPC7 cell line (see Supplementary Fig. 10), consistent with the notion that the relatively small numbers of Runx1 peaks in the ES-cell derived populations are at least in part related to smaller amounts of Runx1 protein available for binding.

We next analysed Runx1 binding peak locations relative to gene coordinates, which demonstrated that the majority of peaks were not associated with promoters but instead found either within or between genes. This pattern of distribution was particularly evident for those peaks already bound in populations 2 and/or 3 where only 3 out of 100 peak regions were found to overlap promoter sequences (see Fig. 3C). By contrast, the proportion of promoter to non-promoter peaks was nearly even for those Runx1 peak regions bound specifically in population 4.

**Fig. 4:**
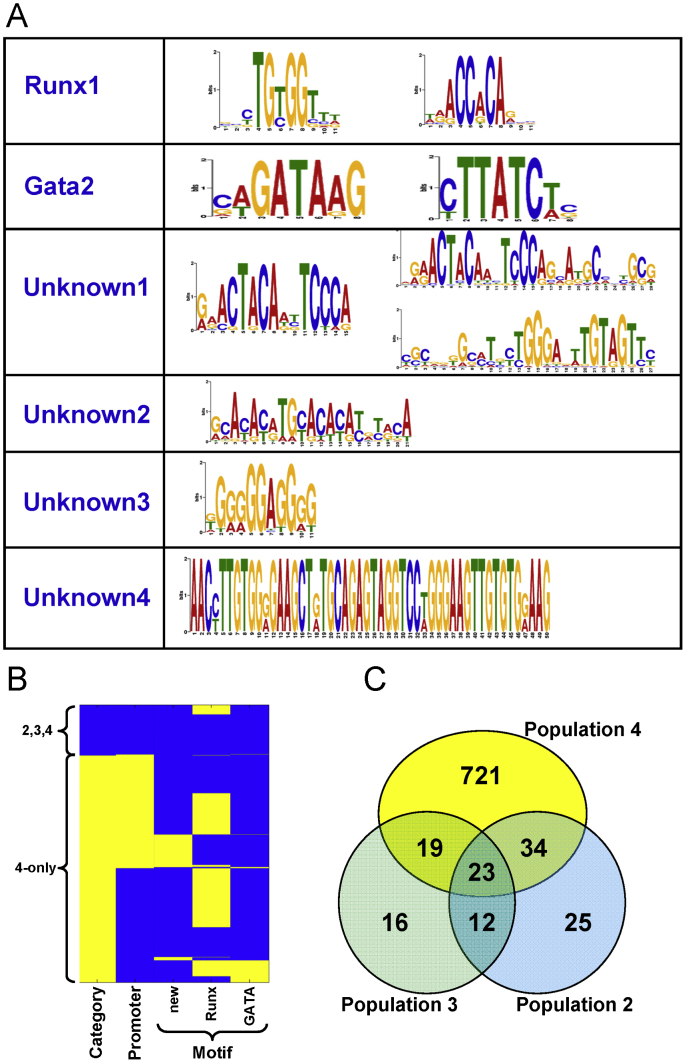
Motif discovery reveals a novel motif enriched in promoters. (A) *De novo* motif discovery reveals three overrepresented motifs with their reverse compliment in Runx1-bound regions, two of which correspond to the Runx and GATA consensus motifs. (B) Hierarchical clustering of all regions bound by Runx1 based on binding profile partitions (populations 2–4 versus population 4 only, location in promoters (second column), and presence of the new, Runx1 or Gata motif from (C) as determined using TFBSSearch (Chapman et al., 2004). (C) Venn diagram of peak overlaps of peaks for the three populations specifically highlighting high overlap between populations 2 and 3 (see also Supplementary Fig. 2).

To explore the nature of Runx1-bound regions further, we performed *de novo* motif discovery to identify overrepresented sequence motifs when compared with control sequences. The most significantly overrepresented motif (see Fig. 4A) corresponded to a perfect match to the Runx1 consensus binding sequence TGYGGT, thus providing independent confirmation of the ChIP-Seq data quality. One of the two further overrepresented motifs was a perfect match to the WGATAA consensus sequence for the GATA family of transcription factors consistent with a recent report suggesting that Gata2 may recruit Runx1 to target regions lacking Runx1 consensus motifs (Wilson et al., 2010a). This notion is supported further by the significant overlap (*P*-value −2.0e^−31^) between the Runx1 targets defined here and Gata2 target sites identified previously in the HPC cells (Supplementary Fig. 5). Moreover, of 1138 WGATA motifs present in the Runx1-bound regions, 403 were situated in an extended WGATAA consensus (expected—1138/4≈285), thus suggesting that A is commonly found immediately 3′ of the GATA core in our data similarly to what has been seen before in erythroid cells (Fujiwara et al., 2009). The final overrepresented motif contained a longer consensus sequence, which contained within its 3′ portion the sequence TGTAGT and therefore deviates from the TGTGGT Runx1 consensus by only a single mismatch. This motif occurs in 10% (79) of all Runx1-bound regions and a very similar motif was recently reported in a ChIP-Seq survey of GABPα binding in human blood stem/progenitor cells (Yu et al., 2011).

To investigate potential dependencies between the various Runx1 binding peak characteristics identified above, we generated a binary table cataloguing peak annotation, and then performed hierarchical clustering. This analysis emphasised our observation that Runx1 binding to promoters is rare for regions bound in populations 2–4, yet relatively common for regions bound in population 4 only (see Fig. 4B). Moreover, even though the Runx1 consensus motif is highly overrepresented and therefore readily recovered by *de novo* sequence motif discovery, it was only present in just over half of all the regions bound by Runx1 suggesting binding via novel motifs or recruitment by other factors. Of note, the novel TGTAGT containing motif (see above) was almost entirely restricted to regions that did not contain the conventional Runx1 consensus motif thus consistent with the notion that it may represent a potentially new motif for Runx1. Moreover, this new motif was found much more often in promoter than non-promoter peaks (*P*-value: 4.8e^−72^) suggesting that Runx1 binding to the TGTAGT site may be facilitated through simultaneous binding of a promoter-specific factor to the adjacent TGGGA sequence, that was also highly conserved within this novel motif (see Fig. 4A,B).

Fig. 4C highlights a high overlap between Runx1-bound regions in populations 2 and 3 providing a rationale for classifying peaks into two partitions (‘2,3,4’ and ‘4 only’) (see Supplementary Fig. 2). This partition was further supported through Pearson correlation analysis of peak height, since ‘2,3,4’ peaks have comparable peak heights in all three populations where as ‘4 only’ peaks are substantially higher in population 4 than in populations 2 and 3. This observation suggests that increased expression of Runx1 in population 4 does not increase the binding strength at previously occupied locations (‘2,3,4’ peaks) but results in Runx1 binding to new genomic locations (‘4 only’ peaks) (see Supplementary Fig. 11). Importantly, *de-novo* motif discovery algorithms revealed overrepresentation of the Runx1 consensus motif in the Runx1-bound regions for each population thus validating both the ‘2,3,4’ and ‘4 only’ peak sets (see Supplementary Fig. 3).

### Integration of expression and ChIP-Seq datasets defines a candidate Runx1 target gene set

**Fig. 5:**
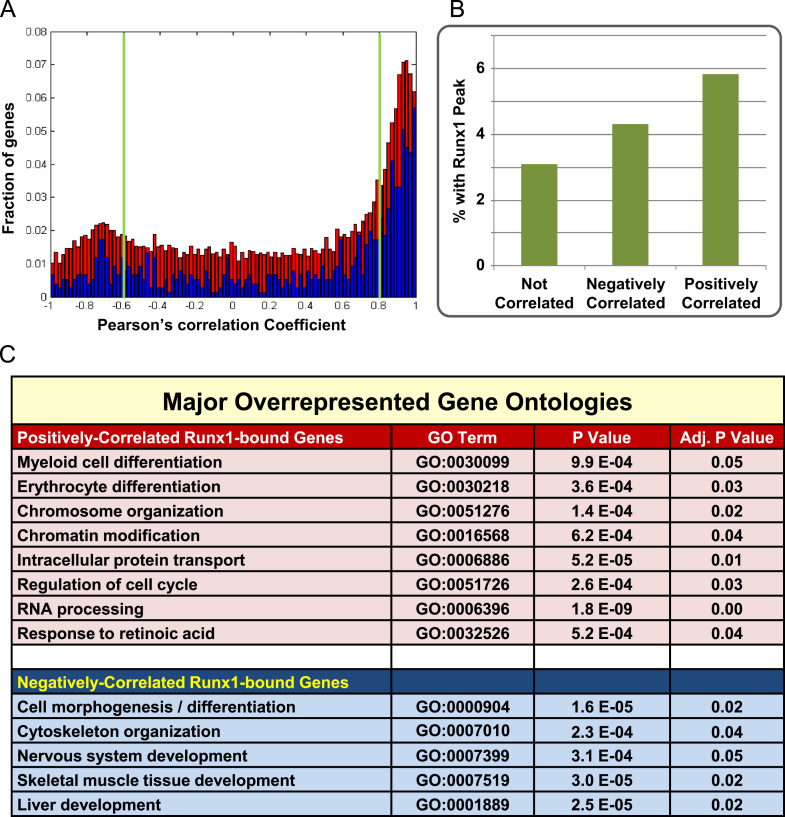
Integration of expression and ChIP-Seq datasets defines a candidate Runx1 target gene set. (A) Histogram showing correlation with Runx1 dynamics for all genes (red) and Runx1 bound genes (blue) (scaled appropriately for superposition) with correlation (0.8) and negative correlation (−0.6) cut-offs. (B) Fraction of Runx1 bound genes in not-correlated, negatively correlated and correlated gene- sets respectively (as defined using the cut-offs in (A). The gradual increase supports the regulatory role of Runx1 inferred from binding events. (C) Analysis of putative gene functions using gene ontology overrepresentation (Al-Shahrour et al., 2008) reveals biological functions consistent with haematopoietic development for correlated Runx1-bound genes, and non-haematopoietic fates for negatively correlated Runx1 bound genes. See Supplementary Fig. 3 for Gene Ontology analysis results using peak regions from single populations.

To delineate the candidate target gene set likely to be directly responsible for mediating Runx1 function during early haematopoietic development, we next performed an integrated analysis of the expression and ChIP-Seq datasets. We mapped the Runx1 binding peaks from all three populations (population 2, population 3 and population 4) to genes which yielded a total combined set of 839 candidate targets (see Supplementary Table 2). We then calculated Pearson correlation coefficients for all 839 candidate targets relative to Runx1 expression across populations 1 to 4. This analysis demonstrated strong enrichment for genes correlating with Runx1 expression, and also a minor enrichment for genes with negatively correlated expression (see Fig. 5A). Moreover, when analysed as a fraction of all expressed genes, those genes that correlated with Runx1 expression were twice as likely to have a Runx1 ChIP-Seq binding peak, than those not correlating with Runx1 expression (see Fig. 5B). However, only approximately 6% of all correlated genes had a binding peak suggesting that only a relatively small subset are direct Runx1 targets. The Runx1 ChIP-Seq data therefore appear to provide a powerful filter for Runx1 target gene identification by reducing candidate targets from over 2900 based on correlated expression alone to just 219 based on the combination of correlated expression and Runx1 binding. Similarly, analysis of the expression data alone had yielded over 1100 negatively correlated genes, yet only 65 of these were classified as direct targets based on the combination of Runx1 ChIP-Seq peaks and negatively correlated expression.

To further explore the potential functionality of Runx1 candidate target genes defined above, we next performed Gene Ontology analysis to identify biological functions overrepresented in the 219 Runx1-bound correlated and also in the 65 Runx1-bound negatively correlated genes (gene lists are provided in Supplementary Table 2). As shown in Fig. 5C, both myeloid and erythroid differentiation were overrepresented in the Runx1-bound positively correlated gene set, as well as a number of additional biological functions including chromatin modification and RNA processing (see Supplementary Fig. 7 for full Gene Ontology tree). By contrast, non-haematopoietic developmental processes (muscle, neural and liver) were overrepresented in the Runx1-bound negatively correlated gene-set, together with biological functions related to cell morphogenesis (see Supplementary Fig. 7 for full Gene Ontology tree). Taken together therefore, Gene Ontology analysis is consistent with the notion that Runx1 upregulated genes drive haematopoietic development, while Runx1 simultaneously represses genes associated with non-haematopoietic tissue development.

### Intersection of the Runx1 candidate target gene set with Runx1^−/−^ expression profiling suggests step-wise establishment of the haematopoietic transcriptional programme

**Fig. 6:**
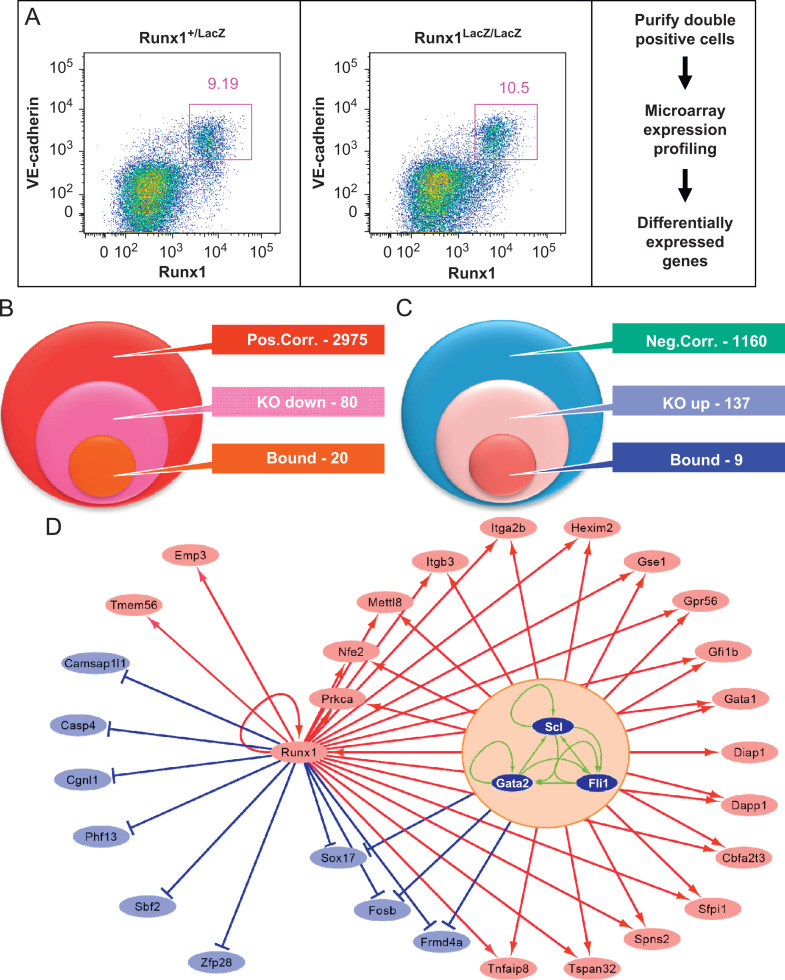
Intersection of Runx1-correlated and Runx1-bound genes with Runx1^−/−^ expression profiling suggests a step-wise establishment of the haematopoietic transcriptional programme. (A) Identification of differentially expressed genes in E7.5 Runx1^+/−^ and Runx1^−/−^ embryos. VE-cadherin^+^/Runx1^+^ cells were flow sorted from E7.5 mouse embryos and differentially expressed genes were identified by microarray expression profiling. The pink square indicates the gate used for cell sorting. (B) Intersection of gene expression and ChIP-Seq datasets identifies a candidate gene set positively controlled by Runx1. ‘Pos.Corr.’, genes positively correlating with Runx1 expression from Fig. 5(A) (e.g., using expression profiling data from *in vitro* differentiated ES cells); ‘KO down’, genes at least 1.5-fold lower in E7.5 Runx1^−/−^ than Runx1^+/−^ from (A) (e.g., using primary cells derived from mouse embryos); ‘Bound’, genes bound by Runx1 in one or more of the three populations from Fig. 3C) Intersection of gene expression and ChIP-Seq datasets identifies a candidate gene set negatively controlled by Runx1. ‘Neg.Corr.’, genes negatively correlating with Runx1 expression from Fig. 5(A) (e.g., using expression profiling data from *in vitro* differentiated ES cells); ‘KO up’, genes at least 1.5-fold higher in E7.5 Runx1^−/−^ than Runx1^+/−^ from A (e.g., using primary cells derived from mouse embryos); ‘Bound’, genes bound by Runx1 in one or more of the 3 populations from Fig. 3D) The majority of Runx1 positively controlled genes from Fig. 6B are bound by the Scl/Gata2/Fli1 triad in haematopoietic progenitor cells. The diagram shows all Runx1-positively controlled genes in red and negatively controlled genes in blue. Links are also drawn for those genes bound by the Scl/Gata2/Fli1 triad in haematopoietic progenitor cells, showing binding for 18/20 Runx1 positively controlled and 3/10 Runx1-negatively controlled genes. Only one of the genes (Mettll3) has a Runx1 peak bound in populations 2–4.

Having identified a core set of Runx1 candidate target genes during the early stages of haematopoietic development based on correlated expression and ChIP-Seq peaks, we next investigated the relationship of this target gene set with Runx1 loss of function expression profiling data. To this end, we purified RNA from flow sorted VE-cadherin^+^/Runx1^+^ cells obtained from E7.5 Runx1 heterozygous and Runx1 null embryos, and performed microarray gene expression profiling to identify all genes affected by loss of Runx1 (Fig. 6A). Using a fold expression change threshold of 1.5, 311 genes showed reduced expression in the Runx1^−/−^ cells, whereas 602 genes were upregulated (see Supplementary Table 3). Since both analyses of Runx1-correlated expression as well as expression changes in Runx1^−/−^ cells will capture both directly and indirectly controlled genes, we next intersected both expression datasets with the Runx1 ChIP-Seq data. A total of 80 genes positively correlating with Runx1 expression in ES cell differentiation assays showed reduced expression in E7.5 Runx1^−/−^ cells, and 20 of these were also identified as Runx1 targets in our ChIP-Seq screen (Fig. 6B). Similarly, of 1160 genes negatively correlating with Runx1 expression, 137 were upregulated in Runx1^−/−^ cells from E7.5 embryos, and 9 of these were bound by Runx1 in the ChIP-Seq (Fig. 6C).

In order to provide further evidence that the Runx1 targets reported here represent a high confidence set of target genes, we investigated how many of the genes shown in Fig. 6(B) are dependent on sustained Runx1 expression in fully developed blood stem/progenitor cells. To this end, we compared the Runx1 targets identified here with a gene list recently reported by Cai et al. (2011), who reported gene expression profiles from wild type and Runx1^−/−^ blood stem/progenitor cells isolated from bone marrow of conditional Runx1 knock-out mice . Of note, 7 out of 19 of our bound and positively-correlated (Itga2b, Hexim 2, Tmem56, Runx1, Mettl8, Gfi1b, Gata1) and 3 out of 10 of our bound and negatively-correlated (Camsap1l1, Casp4, Sbf2) showed differential expression, thus demonstrating dependence on sustained Runx1 expression for a high proportion of the Runx1 candidate target genes identified in our study.

The earliest mesodermal fate restriction en route to blood involves the formation of the haemangioblast, which at the molecular level is characterised by a core regulatory circuit composed of the Scl, Gata2 and Fli1 transcription factors forming a densely connected triad replete with positive feedback loops (Pimanda et al., 2007; Narula et al., 2010). Runx1 itself is not expressed at this early stage (Lancrin et al., 2009). To explore further the potential implications of sequential activation of key regulators, we next interrogated recently published ChIP-Seq data from an ES-cell derived haematopoietic progenitor cell line (Wilson et al., 2010a). Of note, 18 of the 20 genes positively controlled by Runx1 were also bound by the Scl/Gata2/Fli1 triad in the haematopoietic progenitor cell line, while the same Scl/Gata2/Fli1 binding was seen for only 3 of the 10 genes negatively controlled by Runx1 (Fig. 6D). A total of 139/687 ‘4-only’ peaks were bound Scl, Gata2 and Fli1, with a further 413 bound by at least 1 factor in the HPC cell line, thus constituting a set of genomic regions well-recognised as transcription factor targets in blood progenitor cells. By contrast, only 6 of the 100 ‘2,3,4’ peaks were bound by Scl, Gata2 and Fli1 in the HPC cell line, with a further 27 bound by at least one factor. This analysis suggests that Scl, Gata2 and Fli1 mediate Runx1 recruitment only to a subset of the ‘2,3,4’ peaks, while they may act in this way for a large proportion of the ‘4-only’ peaks. Such a model would be consistent with our observation that Gata and Ets motifs were overrepresented in the peaks from population 4, but not populations 2 and 3 (see Supplementary Fig. 3). Moreover, we recently reported that the earliest stages of Runx1 expression are independent of Scl, Gata2 and Fli1, thus suggesting Runx1 functions both alone and in conjunction with the Scl/Gata2/Fli1 triad (Kataoka et al., 2011). Taken together therefore, our data suggest a model whereby some early Runx1 targets are expressed independently of the Scl/Gata2/Fli1 triad, but the majority of haematopoietic genes induced by Runx1 will ultimately be controlled by both Runx1 and the Scl/Gata2/Fli1 triad.

### Expression of CD41 correlates with sequential activation of core regulatory circuits

**Fig. 7:**
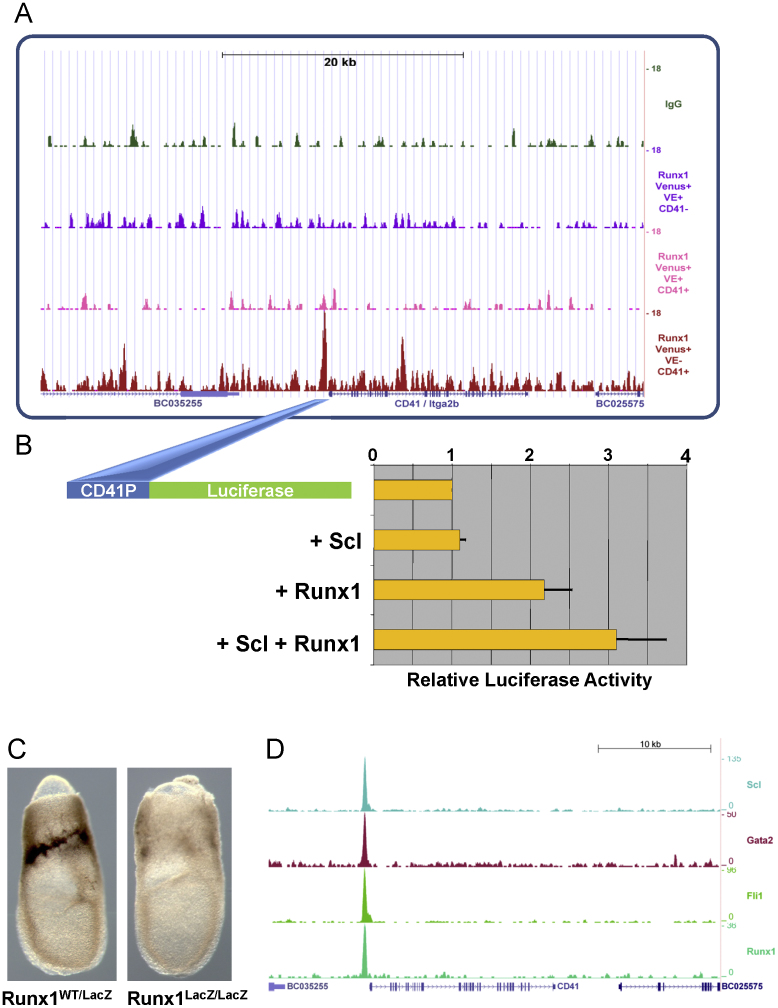
Validation of CD41 as a direct target of Runx1 during early haematopoietic development. (A) Runx1 is recruited to the CD41 promoter during haematopoietic development. Raw ChIP-Seq read data were transformed into density plots and displayed as custom tracks within the UCSC genome browser above the UCSC track for gene structure (IgG control at the top followed by Runx1 data for populations 2–4). (B) The CD41 promoter can be activated by Runx1 and Runx1 together with Scl. The murine CD41 promoter region was inserted upstream of a luciferase reporter gene, and its activity monitored following co-transfection with Scl and Runx1 expression constructs in 293T cells. Relative luciferase activity values are normalised to empty vector control transfections. Values shown represent the average of 3 independent experiments, each performed in triplicate. (C) Early CD41 expression in mouse embryos critically depends on Runx1 activity. Immunostaining for CD41 in Runx1 heterozygous and Runx1 homozygous knock-in E7.5 embryos shows loss of CD41 expression in the absence of Runx1. (D) Scl, Gata2, Fli1 and Runx1 bind to the CD41 promoter in the murine haematopoietic progenitor cell line HPC7. ChIP-Seq results are displayed as density profile custom tracks within the UCSC genome browser as for A.

The integrin alpha 2b gene also known as glycoprotein IIb or CD41 was one of the genes identified in the previous section as bound by both the SclGata2/Fli1 triad and Runx1 in haematopoietic progenitor cells, yet correlating in expression specifically with Runx1 and therefore not expressed as early as Scl. Closer inspection of the Runx1 ChIP-Seq data across the CD41 gene locus demonstrated specific recruitment of Runx1 to the CD41 promoter region with robust Runx1 binding seen in population 4 (see Fig. 7A). CD41 is not expressed in the earlier haemangioblast populations that express the Scl/Gata2/Fli1 triad, yet the CD41 promoter is bound by those three factors as well as Runx1 at the later haematopoietic progenitor stage (see Fig. 7D).

To further investigate the responsiveness of the CD41 promoter to Scl and Runx1, we generated a CD41–promoter construct fused to the luciferase reporter gene and performed transactivation experiments. As shown in Fig. 7(B), transactivation with Scl did not enhance activity of the CD41 promoter. By contrast, Runx1 was able to enhance CD41 promoter activity 2-fold, and this enhancement was even greater (approximately 3-fold), when both Runx1 and Scl were expressed. Differential responsiveness of the CD41 promoter to Runx1 and Scl was therefore consistent with a critical role for Runx1 in initiating CD41 expression, whereas both Runx1 and Scl might collaborate in the maintenance of CD41 expression. Furthermore, Runx1-mediated transactivation of the CD41 promoter was corroborated further since it followed a dose-response pattern (Supplementary Fig. 8).

**Fig. 8:**
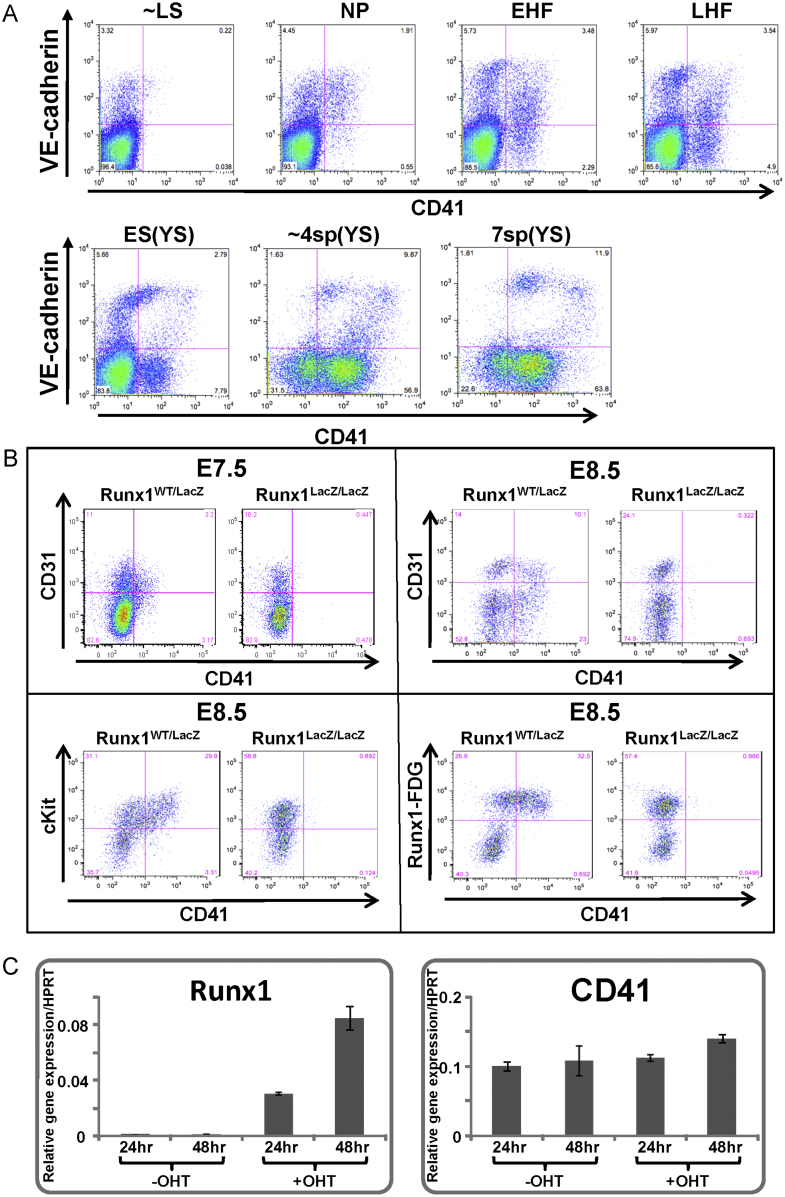
(A) Time course analysis of CD41 expression in early mouse embryos. (A) CD41 and VE-cadherin expression were analysed by flow cytometry between E7.0 and E8.25 of mouse embryonic development. Seven distinct developmental stages between E7.0 and E8.5 were analysed. A distinct CD41 bright population only becomes clearly separable by FACS at the ES stage. LS=late streak stage, NP=neural plate stage, EHF=early head fold stage, LHF=late head fold stage, ES=early somite pair stage (0–1 somite pairs), 4sp=approximately 3–4 somite pair stage, 7sp=7 somite pair stage. The Runx1^+/−^ and Runx1^−/−^ embryos analysed in Fig. 6 are approximately equivalent to the NP stage. Each FACS plot of LS–LHF stage embryos is from 8–10 pooled stage-matched whole embryos. Each FACS plot from somite pair stage embryos is from 4–6 pooled stage-matched yolk sacs. All embryos are from females crossed with GFP-transgenic males to avoid contamination of maternal cells. (B) FACS analysis of E7.5 and E8.5 Runx1-Venus knock-in heterozygous and homozygous mouse embryos. Embryos were dissected and carefully staged. Dissociated cells were analysed by FACS using antibodies against CD41 in combination with anti-CD31 and cKit as well as Runx1-Venus fluorescence. Homozygous Runx1 knock-out embryos show a severe reduction in CD41 positive cells, and a complete absence of cKit-high/CD41^+^ cells. (C) Q-RT-PCR analysis of Runx1 reactivation. Sorted day 4 Flk1+ cells from OP9 co-culture of Runx1-reactivable ES cells (Runx1^SACRE/LacZ^ ES cells) were replated on collagen type IV dishes with or without 4OH-tamoxifen. Cells were harvested for RNA isolation after 24 h and 48 h (see Supplementary Fig. 9 for detailed experimental outline).

To obtain *in vivo* evidence for the proposed critical role of Runx1 in initiating CD41 expression, we next investigated CD41 expression in Runx1 mutant embryos. When we analysed E7.5 wild-type mouse embryos by whole mount immunostaining using antibodies against CD41, CD41 expression was observed within the haematopoietic domain of extraembryonic tissue destined to form the yolk sac blood islands. By contrast, CD41 immunostaining was significantly reduced in Runx1 mutant embryos (see representative embryos shown Fig. 7C). To corroborate these findings, we next performed flow cytometric analysis of both wild type and mutant E7.5 mouse embryos using antibodies against CD41 as well as the endothelial marker CD31. It had previously been reported that E7.0 mouse embryos contain a CD41-dim population, whereas E8.25 embryos contain both CD41-dim and CD41-bright populations (Ferkowicz et al., 2003). We therefore performed extensive FACS analysis of 7 distinct and consecutive developmental stages between E7.0 and E8.5 (see Fig. 8A), in order to interpret analysis of Runx1 embryos within the context of the dynamic changes in CD41 expression. Analysis of E7.5 embryos (where most CD41 expressing cells are CD41-dim) showed that CD31 expression was unaffected in Runx1 mutant embryos, whereas CD41 expressing cells were severely reduced (Fig. 8B). Analysis of E8.5 embryos (where some CD41 expressing cells are CD41-high) showed that CD31 expression was again unaffected in Runx1 mutant embryos. CD41 expressing cells were again severely reduced, particularly so for CD41-high cells (see Fig. 8B). Flow cytometry analysis therefore corroborated the immuno-histological studies. Moreover, since Runx1 mutant embryos express haemangioblast marker genes including Scl, our experiments provide *in vivo* evidence that the transcriptional programme initiated by Scl is not sufficient to mediate CD41 expression in E7.5 mouse embryos, which instead critically depends on Runx1 activity.

### Upregulation of CD41 expression by Runx1 induction in ES cell differentiation culture

CD41 expression was severely reduced in E7.5 Runx1-null embryos. Chip-Seq analysis and CD41 promoter assays showed that Runx1 bound to the CD41 promoter region and upregulated CD41 expression (see Supplementary Fig. 9 for a schematic representation). Next we examined whether Runx1-reactivation in Runx1-null ES cells was able to upregulate CD41 expression as further confirmation for this regulatory link within an *in vitro* ES cell differentiation culture system. To this end, we established Runx1-reactivable ES cells (Runx1^SACRE/LacZ^ ES cells) from a transgenic mouse model where a Runx1-LacZ null allele can be reactivated by 4-hydroxy-tamoxifen (4OHT) (Samokhvalov et al., 2006; Tanaka et al., 2012). When using wild-type ES-cells, a subset of Flk1^+^ cells start to express Runx1 at around day 4 of ES cell differentiation culture on OP9 (Hirai et al., 2005). Therefore, we sorted Flk1^+^ cells after 4 days of culture on OP9, and then recultured cells on collagen type IV dishes with or without 4OHT. After 24 h and 48 h culture, we collected cells for gene expression analysis, which showed specific induction of Runx1 in the 4OHT treated cells. Subsequent analysis of CD41 mRNA levels showed minor but reproducible upregulation, but nevertheless demonstrated CD41 expression to correlate with Runx1 (Fig. 8C). Moreover, similar results have been reported for Pu.1 using a tetracycline-inducible Runx1-reactivation ES-cell based model (Hoogenkamp et al., 2009), thus further validating our strategy of intersecting ChIP-Seq data with both ES-cell and embryo-derived expression datasets.

## Discussion

Transcription factors are widely recognised as key drivers of early development, where they function as central components of gene regulatory networks that execute the genomic regulatory blueprint for cellular differentiation (Davidson and Levine, 2008). Genetic perturbation experiments of key regulatory transcription factors often result in the failure to develop entire cell lineages. One such critical transition step has been revealed through comprehensive *in vitro* and *in vivo* studies of the Runx1 protein, showing it to be indispensable for the development of blood stem/progenitor cells from haemogenic endothelial cells (Swiers et al., 2010). To understand the molecular mechanisms underlying this key developmental transition, identification of physiological Runx1 target genes in early cell populations will be essential. The genome-wide Runx1-centric expression and DNA binding data reported here represent the first integrated genome-scale analysis of complementary expression and ChIP-Seq profiles for any major haematopoietic regulator during a time-course of early haematopoietic development. Comprehensive bioinformatic analysis combined with experimental assays not only provided new insights into the control of early haematopoietic development by Runx1, but may also serve as a blueprint for future analysis of additional key regulators.

### Gradual Runx1 recruitment to target genes during early development

By generating genome-scale data across four developmentally related cell populations that represent the dynamics of early Runx1 induction, we were able to monitor how Runx1 is recruited to its first physiological target genes. This analysis demonstrated that robust Runx1 binding to most targets was only seen in the fourth and most mature population. While the underlying mechanisms remain to be elucidated, one of the most likely explanations may be that, following the first production of Runx1 protein during development, initial Runx1–DNA interactions may be short-lived and therefore not readily identified by ChIP. Indeed, this notion is supported by the observation that most regions called as peaks only in population 4 already have some enrichment for Runx1 binding in population 3 and some also in the earliest Runx1 expressing population 2 (see Fig. 2A). Short-lived ‘priming’ interactions involving Runx1 have been reported previously in a detailed analysis of Runx1 recruitment to the Pu.1–14 URE enhancer (Hoogenkamp et al., 2009). The genome-scale analysis reported here suggests that gradual recruitment of Runx1 is a widespread phenomenon. Importantly however, 100 Runx1 target regions showed robust binding already in the earlier populations suggesting a diversity of mechanisms for Runx1 recruitment, possibly depending on whether or not a given target region has already been ‘opened up’ by prior binding of other factors. It is likely that future studies with increased sequencing depth will identify additional low-level/short-lived binding events for Runx1 as well as other key transcriptional regulators.

### Identification of physiological Runx1 targets through genome-scale timecourse analysis of early haematopoietic development

Experimental strategies for the identification of transcription factor target genes commonly involve perturbation analysis, where expression changes of candidate target genes are taken as evidence for a regulatory connection with the upstream transcription factor. However, perturbation of Runx1 during early haematopoietic development fundamentally changes the cellular phenotypes of early progenitor cell populations, and therefore represents a potential impediment towards the identification of physiological target genes. We therefore elected to generate genome-wide expression and ChIP-Seq time-course data from normal cells as well as expression data from flow-sorted Runx1^+/−^ and Runx1^−/−^ cell populations from E7.5 early embryos in order to identify a high-confidence Runx1 target gene set.

The validity of the above approach is illustrated by the following points: (i) our high-confidence target gene list included Pu.1/Sfpi1 and Runx1 itself, and therefore contained the two early Runx1 targets known previously. (ii) CD41 had previously been categorised as a direct Scl target gene in ES-cell differentiation assays, yet its dependence on Runx1 activity appeared to differ depending on the type of ES-cell differentiation assay used (Mikkola et al., 2003; Lancrin et al., 2009). The genome-scale data presented here suggested that Runx1 is indeed important for early CD41 expression, which we validated *in vivo* by showing that E7.5Runx1^−/−^ embryos do not express significant levels of CD41 even though they are known to express Scl. (iii) A recent study overexpressing Runx1 in haemangioblasts suggested that Runx1 may repress endothelial genes (Sakai et al., 2009). Interestingly, there was a highly significant overlap (*P*-value 1.7e^−12^) between this study and the Runx1 negatively correlated genes reported here, which included Sox17, ectopic expression of which was recently shown to induce apoptosis in early ES cell-derived blood progenitors (Serrano et al., 2010). Direct repression of Sox17 by Runx1 is therefore consistent with the known function of Runx1 as a positive driver of early haematopoietic specification, and underscores the notion that this important function of Runx1 may include repression as well as activation of target genes.

### Sequential deployment of regulatory circuits during a developmental time course

Recent large scale regulatory network studies in lower model organisms have revealed that tissue-specific gene expression is controlled by densely connected networks of TFs which directly control downstream effector genes rather than operating through extended regulatory cascades (Davidson, 2010; Roy et al., 2010; Wilson et al., 2011). However, it is currently not known how these densely connected multi TF networks are established during the specification of cell types from undifferentiated pluripotent progenitors, which typically do not express any of these major tissue-specific TFs. Of note, expression of the various lineage-specific transcription factors is typically initiated at different time-points during tissue specification. For example, whereas all members of the previously described Scl/Gata2/Fli1 haemangioblast triad are expressed prior to Runx1, we have shown here that other regulators such as Pu.1 and Gfi1b follow the Runx1 pattern. Given that the latter two were also direct Runx1 targets, our results are consistent with a model whereby individual subcircuits are activated sequentially during increasing differentiation towards haematopoietic fate. The mechanisms underlying step-wise activation of the proposed core circuits represent important targets for future investigation, with the recent demonstration of Runx1 repression by HoxA3 in haemangioblasts providing a potentially very significant first clue (Iacovino et al., 2011).

Comparison of genome-scale data during haematopoietic specification (this paper) with data from fully specified blood progenitor cells (Wilson et al., 2010a) demonstrated that many of the early Runx1 targets are targets of both Runx1 and the Scl/Gata2/Fli1 triad in fully specified haematopoietic progenitor cells (see Fig. 4). These genes correlate with Runx1 expression during early haematopoietic specification, thus suggesting that even though they ultimately end up downstream of the Scl/Gata2/Fli1 triad, this triad is not sufficient to initiate their expression, which by contrast appears to require Runx1. This hypothesis was indeed corroborated through our complementary *in vitro* and *in vivo* analysis of CD41 regulation. A model is therefore emerging whereby key regulators or small regulatory core circuits are activated sequentially during development, yet end up controlling target genes collectively. Within a fully specified tissue therefore, developmental regulatory cascades effectively collapse into one large and densely connected TF network, which directly controls expression of tissue specific effector genes.

One important mechanism involved in stabilising these large transcription factor networks is likely to be extensive protein-protein interactions, new examples of which continue to be discovered (Wilson et al., 2010a; Palii et al., 2011) including the recent demonstration of Runx1–Gata2 interactions which would link Runx1 with the earlier haemangioblast core circuit. The resultant crosstalk would have the capacity to stabilise emerging regulatory networks assembled from sequentially activated individual TFs or small core circuits. Validation of this model will require time-course analysis of multiple key regulatory TFs in addition to the data on Runx1 presented here. Nevertheless, the comprehensive genome-scale analysis of Runx1-centric gene expression and ChIP-Seq data reported here illustrates the power of this approach; not only to enhance our understanding of the functionality of an individual regulator, but also to gain potentially widely applicable insights into the transcriptional control of mammalian development.

## Authorship contributions

YT, AJ, NKW and SK performed the work. YT, AJ, SN and BG wrote the paper. SN and BG designed the study.

## Conflict of interest disclosure

The authors declare that there are no conflicts of interest.

## Footnotes

Appendix A Supplementary data associated with this article can be found in the online version at doi:10.1016/j.ydbio.2012.03.024.
