# Supplementary material for: The transcriptional programme controlled by Runx1 during early embryonic blood development
Source: Dev Biol. 2012 Jun 15;366(2):404–19. doi: 10.1016/j.ydbio.2012.03.024 (PMC3430866; doi:10.1016/j.ydbio.2012.03.024)
Supplement: Supplementary file 1 — Supplementary material [file mmc1.pdf]

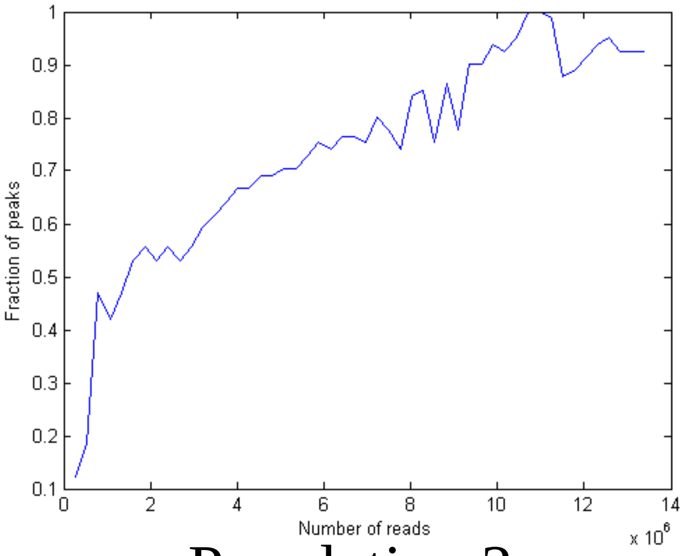

Population 2

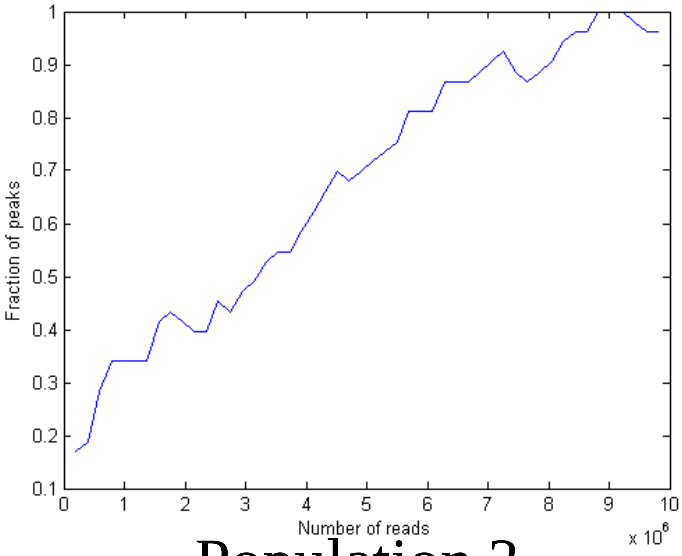

Population 3

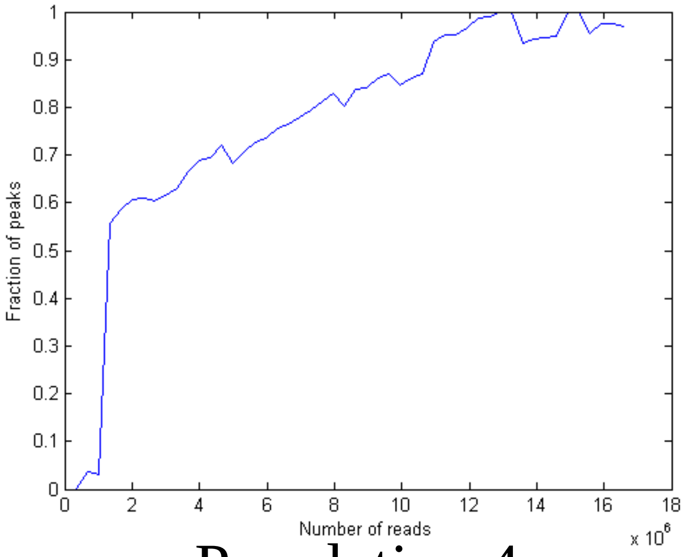

Population 4

Supplementary figure 1:
Saturation curves for Runx1 ChIP-Seq peak calling in three ES-cell derived populations (populations 2,3 and 4).
Random subsets of total reads were selected and peaks called using MACS. Fraction of peaks recovered from each subset shows that all 3 populations approach a plateau, thus suggesting sufficient sequencing depth for meaningful peak calling.

Supplementary Figure 1

A

	Population 2	Population 3	Population 4
Population 2	1.0	0.83	0.35
Population 3	0.83	1.0	0.15
Population 4	0.35	0.15	1.0

B

	Population 2	Population 3	Population 4
Population 2	1.0	0.83	0.03
Population 3	0.83	1.0	0.08
Population 4	0.03	0.08	1.0

Supplementary figure 2:
Motif and Gene Ontology analysis of Runx1 ChIP-Seq results for individual populations.

A) Pearson's correlation coefficient of Runx1 peak heights (RPKM) in the three ES-cell derived populations for '2,3,4' peaks (100 peaks).
B) Pearson's correlation coefficient of Runx1 peak heights (RPKM) in the three ES-cell derived populations for '4 only' peaks (687 peaks).

Supplementary Figure 2

A

	Peak Numbers	Sequence motifs
Population 2	67	Max, JunD, CRE, Runx
Population 3	51	Runx
Population 4	747	Runx, Gata, ETS,JunD

B

	GO overrepresentation (p value < 0.001)
Population 2	Chitin binding, membrane, ABC transporters
Population 3	Chitin binding, membrane, ABC transporters, cytosceleton
Population 4	Phosphoprotein, acetylation, transcription regulation, myeloid cell differentiation, endomembrane system

Supplementary figure 3:
Motif and Gene Ontology analysis of Runx1 ChIP-Seq results for individual populations.
A) Overrepresented motifs in Runx1 ChIP-Seq peaks from populations 2, 3 and 4. Shown is the number of peaks found for each population as well as the names of overrepresented motifs.
B) Gene ontology functional enrichment analysis for genes next to Runx1 ChIP-Seq peaks from populations 2, 3 and 4. Shown are all Gene Ontology categories enriched with a p value < 0.001.

Supplementary Figure 3

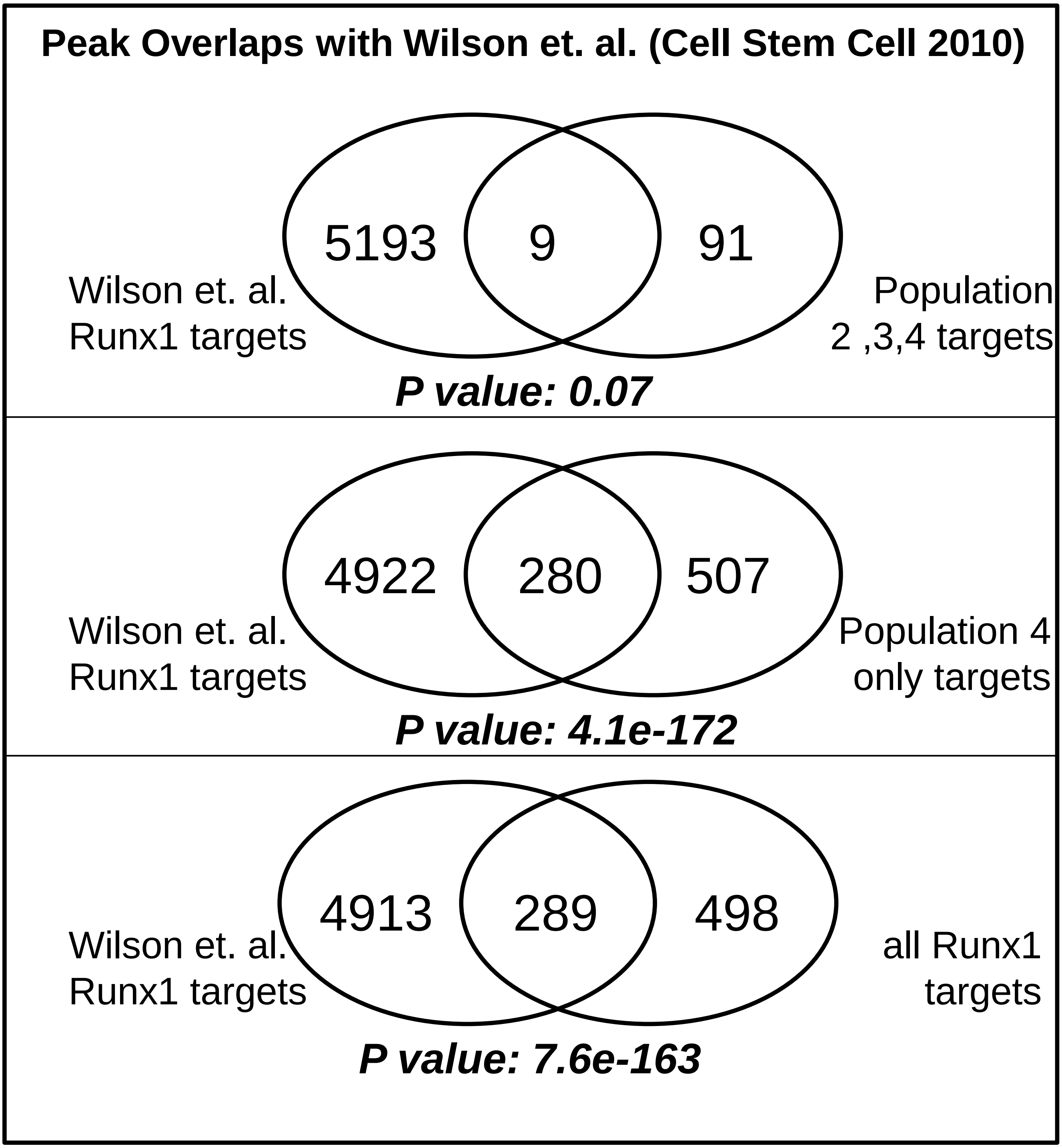

Supplementary figure 4:
Comparison of Runx1 ChIP-Seq targets from three ES-cell derived populations with Runx1 ChIP-Seq targets in HPC7 cells.
Venn diagrams show peak overlaps of Ruxn1 targets of Wilson et. al. (Cell Stem Cell, 2010) and Runx1 targets in “population 2,3,4”, “population 4” and “all Runx1” targets combined.

Supplementary Figure 4

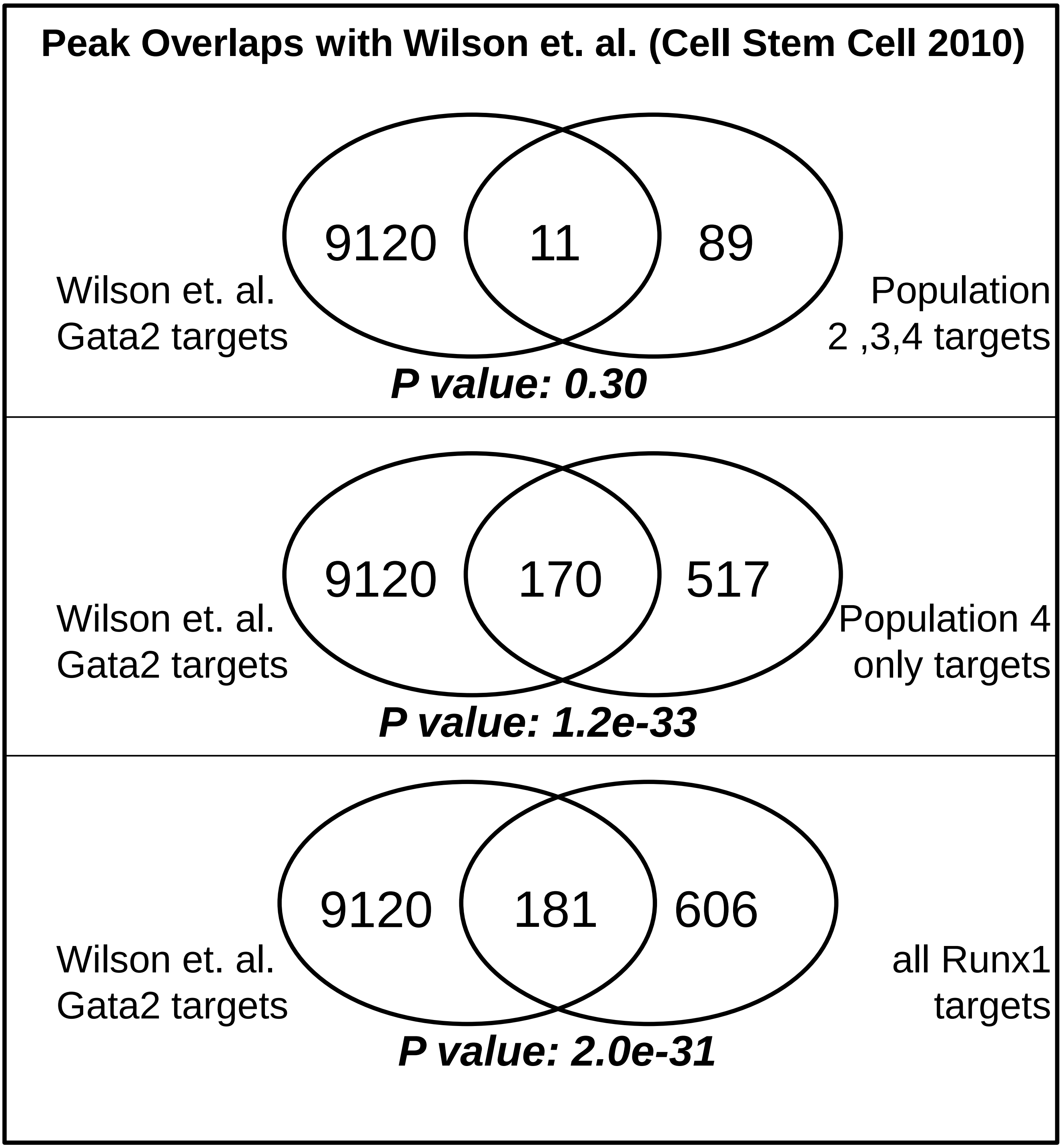

Supplementary figure 5:
Comparison of Runx1 ChIP-Seq targets from three ES-cell derived populations with Gata2 ChIP-seq targets in HPC7 cells.

Venn diagrams show peak overlaps of Gata2 targets of Wilson et. al. (Cell Stem Cell, 2010) and Runx1 targets in “population 2,3,4”, “population 4” and “all Runx1” targets combined

Supplementary Figure 5

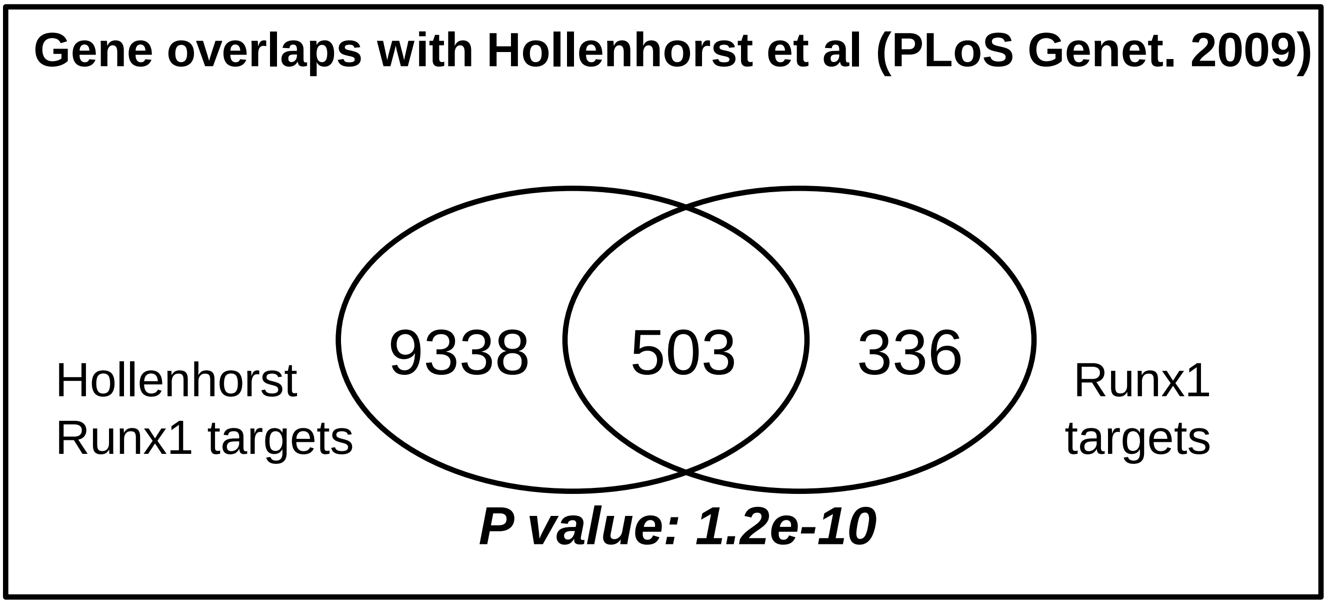

Supplementary figure 6:
Comparison of Runx1 ChIP-Seq targets from three ES-cell derived populations (Mouse) with Runx1 ChIP-Seq targets in Jurkat T cells (Human).
Venn diagram shows gene overlap of Runx1 targets of Hollenhorst et. al., 2009 and all Runx1 targets identified in he current study.

Supplementary Figure 6

A

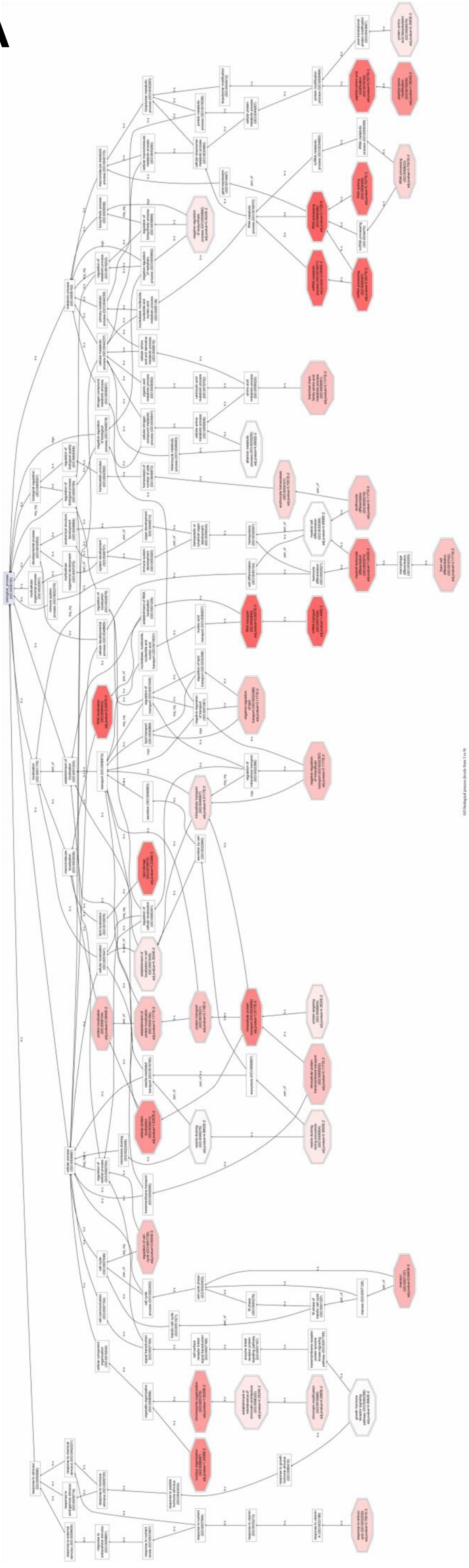

B

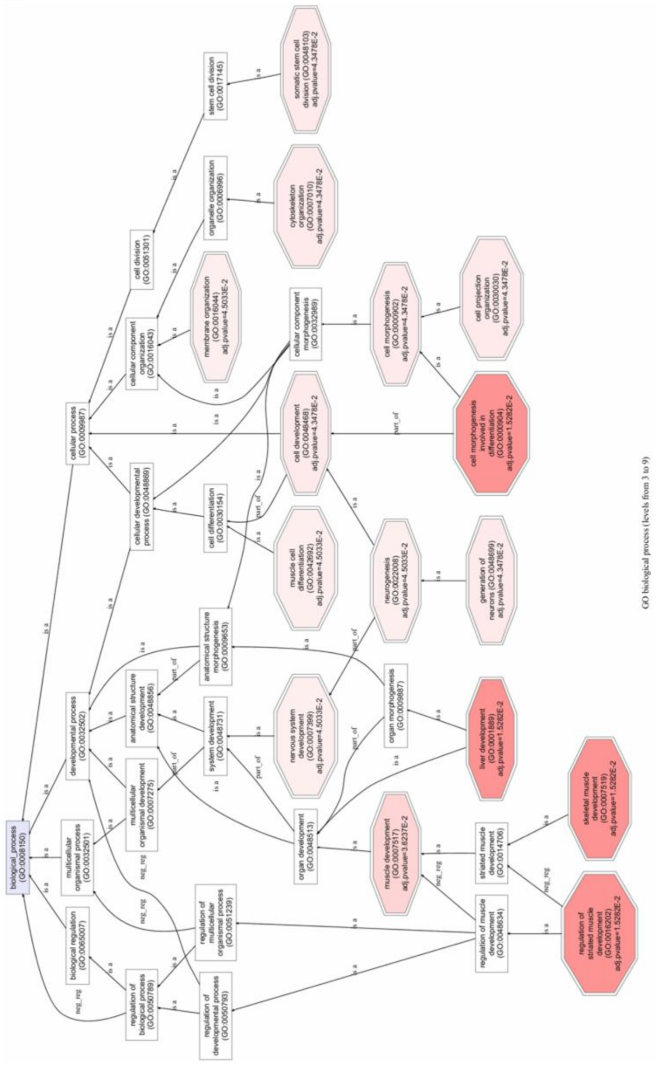

Supplementary Figure 7: Gene ontology (GO) analysis for Runx-1 bound correlated (A) and anti-correlated (B) genes.

Shown are the full GO trees generated by the FATIGO program as part of the BABELOMICS suite of analysis tools (Medina et al, Nucleic Acids Res. 2010. 38(Web Server issue):W210-3). The degree of shading corresponds to the significance of overrepresentation.

Supplementary Figure 7

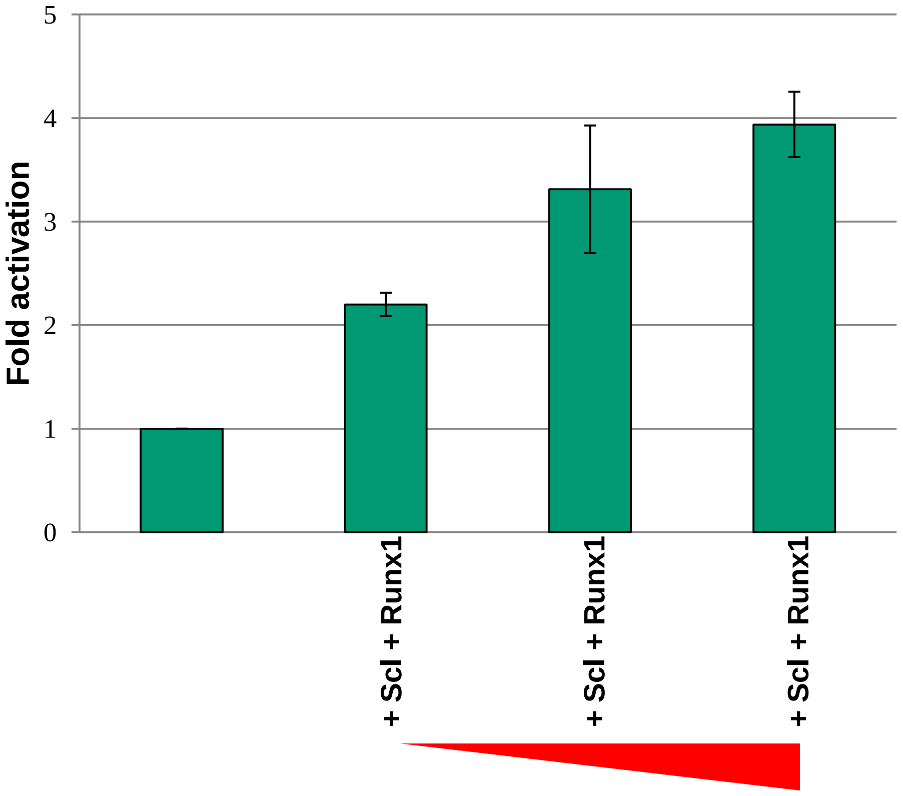

Supplementary Figure 8:

The CD41 promoter was co-transfected with increasing amounts of Runx1 and Scl. Increasing amounts of Scl and Runx1 resulted in an increasing level of transactivation. Relative luciferase activity values are normalised to empty vector controls. Values shown represent the average of 2 independent experiments each performed in triplicate.

Supplementary Figure 8

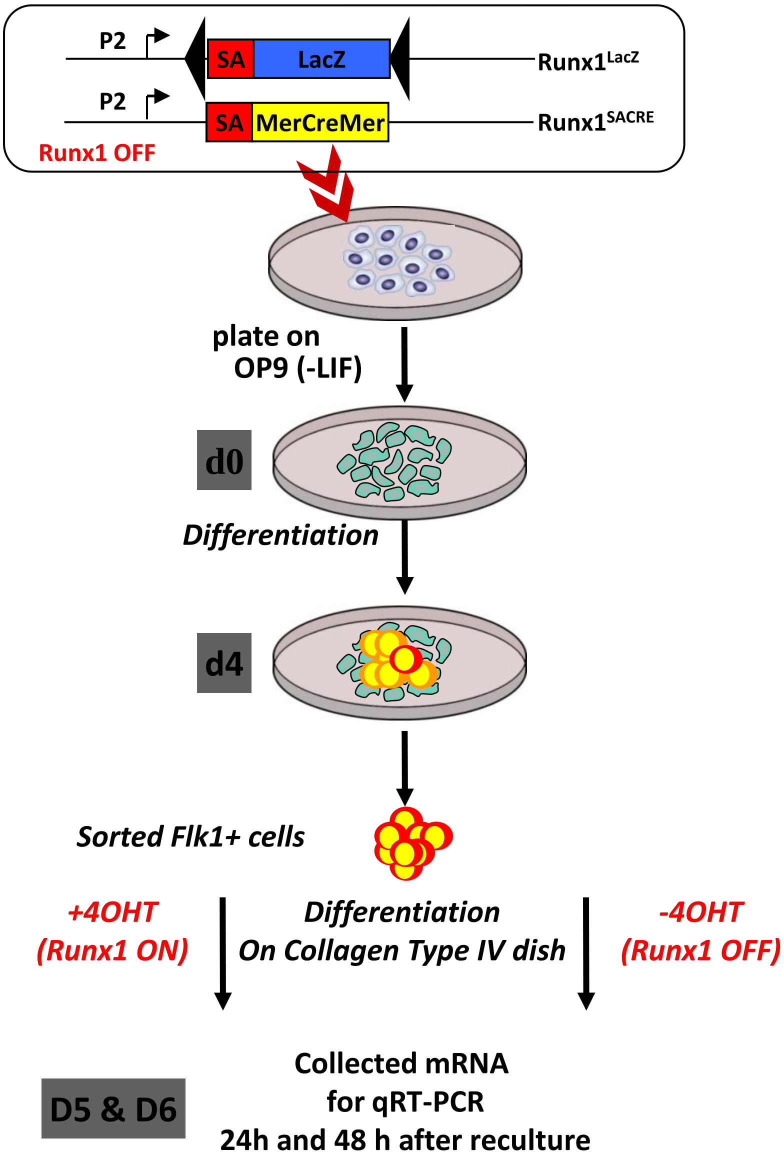

Supplementary Figure 9:
Experimental design for Runx1-reactivation
ES cells were generated carrying a splice acceptor followed by an estrogen receptor Cre recombinase fusion (SA-MerCreMer) in one Runx1 allele, and a loxP-flanked lacZ in the other, with the latter representing a conditionally reactivatable allele. Runx1$^{SACRE/LacZ}$ ES-cells were differentiated on OP9. At day4 of differentiation, Flk1+ cells were sorted and recultured on collagen type IV dishes with or without 4 Hydroxy Tamoxifen (4OHT). After 24h and 48 h of reculture, cells were harvested and collected for RNA isolation.

Supplementary Figure 9

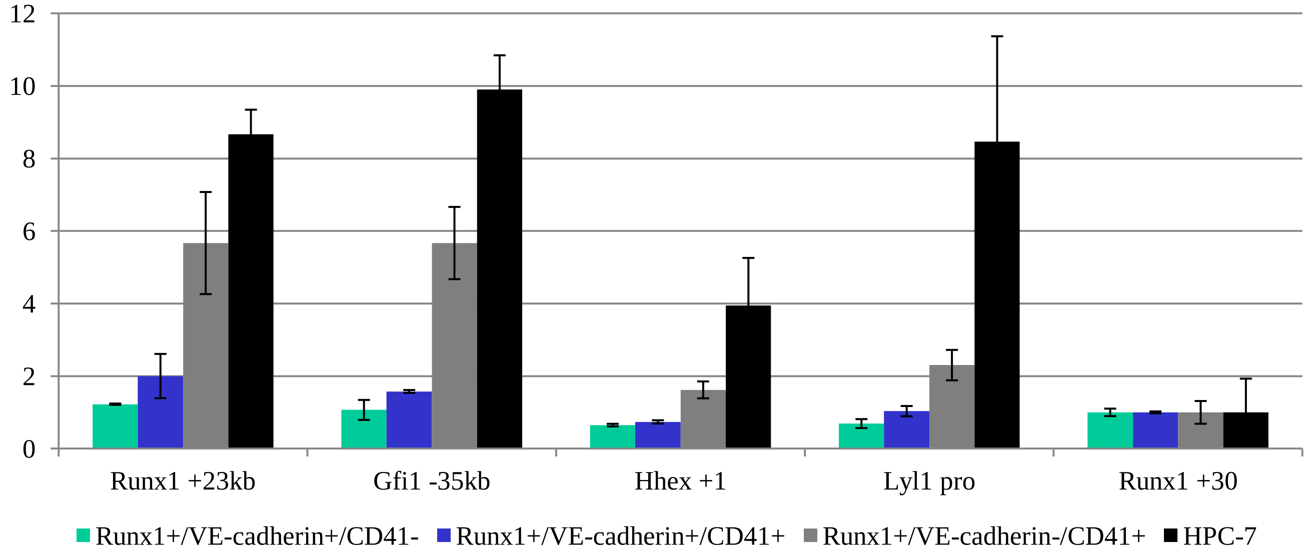

Supplementary Figure 10:

ChIP was performed in HPC-7 cells using an equivalent cell number to that used for the three ES-cell derived populations. Quantitative real time PCR was performed to quantify the binding of Runx1 at known targets of Runx1 binding in haematopoietic stem/progenitor cells. Runx1 binding was normalised to IgG control and to a negative region (Runx1 +30kb).

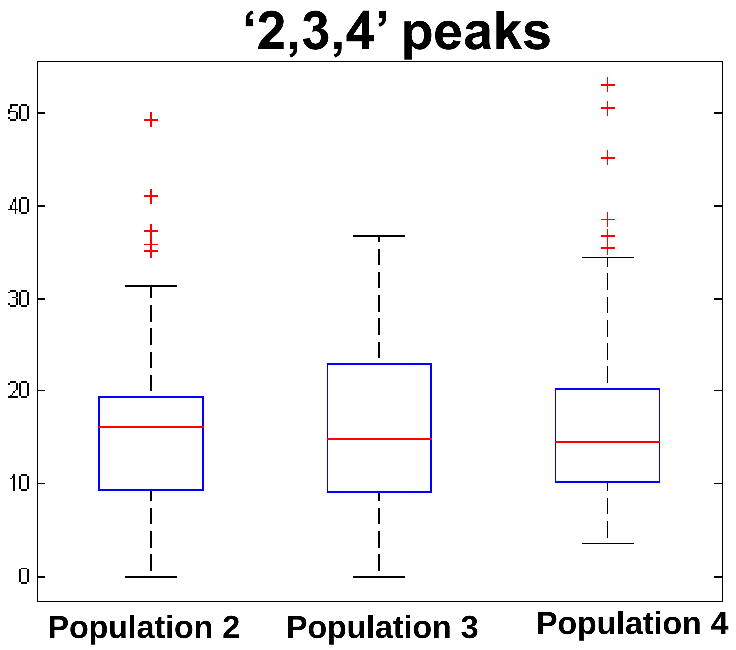

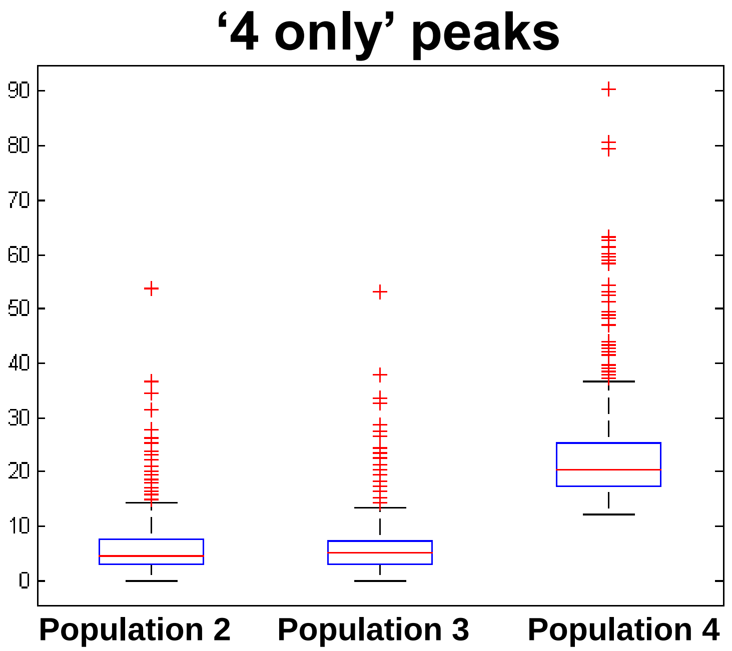

Supplementary figure 11:
Box plots of peak heights for peak partitions ‘2,3,4’ and ‘4 only’

‘2,3,4’ peaks have similar Runx1 ChIP-Seq peak heights in all 3 populations while ‘4 only’ peaks show high peak heights in population 4 compared to populations 2 and 3.

Supplementary Figure 11
